# Maritime trap-jaw ants (Hymenoptera, Formicidae, Ponerinae) of the Indo-Australian region – redescription of *Odontomachus malignus* Smith and description of a related new species from Singapore, including first descriptions of males

**DOI:** 10.3897/zookeys.915.38968

**Published:** 2020-02-24

**Authors:** Wendy Y. Wang, Aiki Yamada, Seiki Yamane

**Affiliations:** 1 Lee Kong Chian Natural History Museum, National University of Singapore, 2 Conservatory Drive, 117377, Singapore National University of Singapore Singapore Singapore; 2 Systematic Zoology Laboratory, Department of Biological Sciences, Graduate School of Science, Tokyo Metropolitan University, 1-1 Minami-Osawa, Hachioji-shi, Tokyo, 192-0397, Japan Tokyo Metropolitan University Tokyo Japan; 3 Kagoshima University Museum, Kôrimoto 1-21-30, Kagoshima 890-0065, Japan Kagoshima University Museum Kagoshima Japan

**Keywords:** inter-tidal ants, littoral habitat, mangroves, systematics, sympatric species

## Abstract

The maritime trap-jaw ant *Odontomachus malignus* Smith, 1859 is thought to be widespread throughout islands in the Indo-Pacific and parts of the Oriental realm. Because of its unique nesting preference for harsh littoral habitat and distinct morphology, *O. malignus* has usually been assumed to consist of only one species. We, however, describe a new species similar to *O. malignus* found in the mangroves of Singapore, Southeast Asia – *Odontomachus litoralis***sp. nov**. We find strong evidence of both species existing in (near) sympatry, and also distinct morphological differences between *O. malignus* and the new species. Additional complementary DNA evidence in the form of COI barcodes (313 bp) supporting putative species identification and delimitation is provided. Defining morphological characteristics for the *O. malignus* species group (nested within the larger *O. infandus* clade) are given in detail for the first time. The worker and queen castes of the new species are described; a redescription of the worker caste of *O. malignus*, based on specimens from Singapore and the Philippines in addition to the holotype, is also given. The males of both species are also described for the first time, including male genitalia. A preliminary key to most known species of the *O. infandus* group based on the worker caste is provided.

## Introduction

The trap-jaw ant genus *Odontomachus* Latreille, 1804 (Hymenoptera: Formicidae: Ponerinae) comprises 72 valid extant and three fossil species to date (Bolton 2019), and is widely distributed throughout the tropics, with highest species numbers in the Neotropics and Malesia (Brown 1976). One particular species, *Odontomachus malignus* Smith, 1859, stands out from the rest because of its peculiar habitat preference. This species is exclusively found in one of the harshest and most volatile places in the tropics, the intertidal littoral zone, usually on coral rubble, sometimes far from the coastline, and limestone rockfaces (Mann 1919, Wilson 1959, Brown 1976, Olsen 2009, Sorger and Zettel 2011). Little is known about the ecology and/or reproductive biology of this unusual ant, and how colonies thrive in an unstable intertidal environment subject to daily fluctuations of abiotic conditions.

The taxonomic history of *O. malignus* follows a rather convoluted trajectory. The species is thought to be widespread and not rare across intertidal areas throughout islands in the Indo-Pacific (Matos-Maraví et al. 2018), now considered part of the Oceanian realm (Holt et al. 2013). However, variation has been observed among different populations by many authors, evoking speculations of either geographical variants of a single species, or the implicit possibility that ‘*O. malignus*’ might comprise a species complex. Smith (1859) first described the species from specimens collected by Alfred Wallace from the Aru Islands, Maluku (Indonesia). Subsequently, Roger (1861) described a similar species – *Odontomachus tuberculatus* Roger, 1861 – collected from an unspecified locality in Asia. Emery (1887) later declared *O. tuberculatus* a junior synonym of *O. malignus*, after examining specimens from Sarawak, Sulawesi (Celebes) and New Guinea. Mann (1919) however, revived *O. tuberculatus* from synonymy as a subspecies of *O. malignus*, on the basis of the former’s longitudinally striate mesonotum, as opposed to the transversely striate mesonotum of *O. malignus*. Mann drew his conclusion after examining large suites of workers collected from Graciosa Bay, Santa Cruz Islands (Papua New Guinea) and Simoli, South Malaita (Papua New Guinea). Both series of workers were found in and around crevices of coral rubble on beaches. Forty years on, Wilson (1959) refuted Mann’s (1919) conclusion and again synonymised the subspecies *O. m. tuberculatus* with *O. malignus*, explaining that the orientation of mesonotal striae in Mann’s (1919) nest series from Santa Cruz was highly variable. Consequently, the orientation of mesonotal striae should not be used to distinguish *O. tuberculatus* from other populations of *O. malignus*.

Similar controversy surrounded the form Odontomachus malignus var. retrolatior Viehmeyer, 1914, which is now considered a junior synonym of *O. malignus*. The variety was described by Viehmeyer (1914), upon examination of specimens collected from West Ceram, Maluku (Indonesia) by H. Streesemann, and from his own collection from Monumbo [sic], New Guinea. Viehmeyer (1914) highlighted many morphological differences that he believed distinguished *O. retrolatior* from *O. malignus*, including: a longer head but less narrowed posteriorly, a shorter petiolar spine, and generally stronger sculpture especially on the occiput and pronotum. Despite the apparent differences, Brown (1976) synonymised *O. retrolatior* under *O. malignus*, citing comparisons of specimens from Alexishafen (Madang, Papua New Guinea) with material from the Philippines in J.W. Chapman’s collection, specifically Tawitawi, Sitanki (Jolo Island, Sulu Archipelago) and Rennell Island.

In addition, Brown (1976) further assigned *O. malignus* to the *O. infandus* species group; this species group is defined mainly by a variable broadening of the vertex, palp formula 4,4, long and acute apical, intercalary and subapical mandibular teeth, and a strong pre-apical series of teeth. Sorger and Zettel (2011) subsequently removed *O. malignus* from the *O. infandus* group based on the former’s distinct morphological and ecological characters; the separate *O. malignus* species group comprising just one eponymous member was erected instead. Using a molecular dataset of 3.8 kb of aligned protein-encoding nuclear and mitochondrial genes, Matos-Maraví et al. (2018) constructed a phylogeny that largely supported the morphological species groups proposed by Brown (1976), including the positioning of *O. malignus* within the *O. infandus* clade. It is notable that an unidentified species from Sarawak (Borneo, Malaysia) was found to diverge from *O. malignus* at an internal node near the tips of the consensus tree (albeit with low support), suggesting a relatively recent-diverged species closely related to true *O. malignus*. Given the molecular evidence presented by Matos-Maraví et al. (2018), we here conservatively treat the *O. malignus* species group as one defined by shared unique morphological and ecological characters sensu Sorger and Zettel (2011) that have not been proven apomorphic, nested within the larger ancestral *O. infandus* clade.

In this study, we describe a new species from Singapore (Southeast Asia) belonging to the *O. malignus* species group, and provide morphological evidence, supported by the existence of workers and males of both species in (near) sympatry and partial molecular evidence (i.e., COI barcodes), showing that the new species is different from *O. malignus*. Based on the limited DNA evidence, we further hypothesise that the new species may confer with the aforementioned unidentified species from Sarawak in Matos-Maraví et al.’s (2018) study. We also identify, based on short fragment DNA barcodes, *O. malignus* males collected in (near) sympatry with the new species from Singapore, and therein provide descriptions and comparisons of the males for *O. malignus* (putative identity based on DNA) and the new species, including male genitalia. The utility of short fragment DNA barcodes as complementary tools in cost-effective species discovery (Wang et al. 2018a, b; Srivathsan et al. 2019) and matching semaphoronts of the same insect species (Yeo et al. 2018) has been shown and validated in recent studies. We further discuss the various bases for distinguishing the two forms as separate species, including morphology, sympatry, nesting ecology, and broad geographic distributions.

## Materials and methods

### Material examined (images)

Type images of the following species and subspecies (synonymised) available at AntWeb v.7.27.2 (Available from https://www.antweb.org. Accessed 14 August 2019) were examined:

“*Odontomachus tuberculatus* Roger, 1861” (Emery 1887, Wilson 1959: junior synonym of *O. malignus*) – syntype, worker, CASENT0915471, Muséum Paris EY9264, Asia – specific locality unknown; antennae missing, mesosoma heavily damaged.

“Odontomachus malignus var. retrolatior Viehmeyer, 1914” (Brown 1976: junior synonym of *O. malignus*) – type, worker, FOCOL1081, Monumbo [sic], New Guinea; type, worker, FOCOL1082, Monumbo, New Guinea; type, worker, FOCOL0402, Monumbo, New Guinea.

### Morphological examination and imaging

Morphological observations of specimens were made using Olympus SZX16 and Nikon SMZ18 stereomicroscopes, while measurements were made using micrometres on the Olympus SZX16. Measurements of the type images provided in AntWeb v.7.27.2 (Available from https://www.antweb.org. Accessed 14 August 2019) were made using ImageJ 1.52c (Schneider et al. 2012; available at http://imagej.nih.gov/ij).

Male genitalia of one male from each species (ZRC_ENT00007636.1, ZRC_BDP0014515), preserved in 80% ethanol, were slide-mounted by following the preparation steps described in Yamada & Eguchi (2016), and examined with a Nikon Eclipse E600 microscope.

Morphological terminology follows mainly Brown (1976) and Satria et al. (2015) with a few modifications (mostly syntactical), and Boudinot (2013) and MacGown et al. (2014) for male genitalia. All measurements are given in millimetres (mm). The abbreviations used for measurements and indices are as follows:

**EL** Compound eye (hereafter simply termed ‘eye’) length measured along its maximum longitudinal diameter with head in lateral view.

**EW** Maximum eye width perpendicular to EL.

**FWL** Maximum forewing length (alate queen and male only).

**HL** Maximum length of head in full-face view, measured from anteriormost point of clypeus to midpoint of a line drawn across posterior margin of head (including ocelli for male).

**HW** Maximum width of head in full-face view. For males, measurement includes compound eyes.

**IFLW** Inter-frontal lobe width, measured as maximum distance between outermost margins of frontal lobe (worker and queen only). The frontal lobe is taken as the median arch of the torulus, as in Keller (2011).

**MDL** Maximum length of mandible measured from mandibular insertion to apex of mandible (worker and queen).

**OL** Ocellus length, measured as maximum diameter of major axis of median ocellus (queen and male).

**OED** Ocello-ocular distance, measured as maximum distance between lateral ocellus and eye (male only).

**PTH** Petiole height, measured as maximum height of petiole in lateral view, perpendicular to petiole length, from an imaginary line tangential to petiolar apex to ventral surface of postpetiolar helcium, where the latter inserts into the petiole. Note: PTH here is measured only to the ventral surface of the postpetiolar helcium, instead of the ventral-most point of the subpetiolar process sensu MacGown et al. (2014) and Satria et al. (2015). This is to ensure fair comparisons of PTH with *O. malignus* specimens (or their images), including the holotype, that are mounted such that the subpetiolar process is obscured or not measurable.

**PTL** Petiole length, measured as maximum length from anteriormost to posteriormost inflections of petiolar node in lateral view.

**SL** Maximum length of antennal scape excluding basal constriction.

**WL** Weber’s length, maximum diagonal distance of mesosoma in lateral view, measured from base of anterior slope of pronotum to posterior-most point of propodeal lobe.

**CI** Cephalic index: HW/HL × 100

**MDI** Mandible index: MDL/HL × 100

**PTHI** Petiole height index: PTH/PTL × 100

**SI** Scape index: SL/HW × 100

### Depositories

**NHMW** Naturhistorisches Museum Wien, Vienna, Austria

**OUMNH** Oxford University Museum of Natural History, Oxford, UK

**SKYC** Seiki Yamane Collection, Kagoshima, Japan

**ZRC** Zoological Reference Collection, Lee Kong Chian Natural History Museum, Singapore

Source images for focus stacking were taken using a Canon EOS Kiss X9 digital camera, attached to a Nikon AZ100 stereomicroscope (for *O. litoralis* worker, queen, and male bodies of all species, excluding male genitalia), and a Nikon Eclipse E600 microscope (for male genitalia). Focus-stacked images were produced using Helicon Focus Pro 7.0.2 (Helicon Soft Ltd., http://www.heliconsoft.com/), and improved with the retouching function of the same software. Colour balance and contrast were adjusted using GIMP 2.8 (The GIMP Development Team, http://www.gimp.org).

Workers of *O. malignus* were imaged with a Dun Inc. Passport II macrophotography imaging system, using a Canon MP-E 65 mm lens; focus-stacked images were produced using Zerene Stacker v1.04 (Zerene Systems LLC, https://zerenesystems.com/cms/stacker). All final images were further adjusted, annotated, and scale bars added using Adobe Photoshop CS6.

### DNA barcoding

DNA barcoding and subsequent objective clustering were conducted to roughly sort specimens into putative molecular taxonomic units for further morphological review. DNA extraction was performed on six individuals (4 workers, 1 queen, 1 male) from the type series of the new species (catalogue numbers ZRC_ENT00000917.01–06) collected in Singapore, and 10 unidentified males collected in (near) sympatry from mangroves in Pulau Semakau (Singapore) using malaise traps (catalogue numbers each with prefix ‘ZRC_BDP’). One leg per individual was used for DNA extraction with QuickExtract DNA extraction solution (Kranzfelder et al. 2016) as per manufacturer’s instruction; the dissected specimens were later dry mounted for morphological examination. PCR amplification of a 313 bp fragment of the mitochondrial gene cytochrome c oxidase subunit I (COI), and next generation sequencing of the amplicons were performed on the Illumina MiSeq platform following procedures described in Wang et al. (2018a). Successful barcodes were checked for contamination and identities against the GenBank (NCBI) nucleotide database (Benson et al. 2013), using the online NCBI Basic Local Alignment Search Tool (BLAST) ver.2.6.0+ (Altschul et al. 1990) under default parameters in *Megablast* (word size: 28).

Barcodes were aligned using MAFFT v7 (Katoh and Standley 2013), and alignments were checked on MEGA 6 (Tamura et al. 2013). We used a custom-built Python script-based software *obj_clust* v 0.1.2 (Srivathsan, A. unpublished; an implementation of objective clustering in SpeciesIdentifier (Meier et al. 2006)) to group sequences according to uncorrected *p*-distances using the ‘best close match’ criteria (Meier et al. 2006, 2008) – members of a set of putative conspecific sequences have at least one match to a sequence in that set, which falls within a given percentage distance threshold. For a rough sorting into putative molecular operational taxonomic units, we performed clustering on barcodes obtained from specimens collected in Singapore, Borneo, Palau, and the Philippines. Sequences from specimens collected outside of Singapore were identified and retrieved from GenBank via the prior BLAST step. We also included in the clustering step, COI sequences from sympatric populations (i.e., from Singapore) of other morphologically verified named congeneric species (*O. rixosus*, *O. pararixosus*, *O. simillimus*) as conceptual references for the utility of objective clustering in rough species sorting. The breakdown of COI sequences used in objective clustering and their respective associated information is shown in Table 1.

**Table 1.** Summary of specimen data associated with COI sequences used in objective clustering.

Species	Specimen identifier/catalogue no.	GenBank accession no.	Geographic origin of specimen	Caste/sex
*O. litoralis*	ZRC_ENT00000917.1	MK910364	Singapore	Worker
	ZRC_ENT00000917.2	MK910365	Singapore	Worker
	ZRC_ENT00000917.3	MK910366	Singapore	Worker
	ZRC_ENT00000917.4	MK910367	Singapore	Worker
	ZRC_ENT00000917.5	MK910368	Singapore	Queen
	ZRC_ENT00000917.6	MK910369	Singapore	Male
	Sp. BOR002, MJ19771	KU146009	Sarawak, Borneo	Worker
*O. malignus*	ZRC_BDP0014432	MK910354	Singapore	Male
	ZRC_BDP0014442	MK910355	Singapore	Male
	ZRC_BDP0014515	MK910356	Singapore	Male
	ZRC_BDP0014516	MK910357	Singapore	Male
	ZRC_BDP0014535	MK910358	Singapore	Male
	ZRC_BDP0014676	MK910359	Singapore	Male
	ZRC_BDP0014677	MK910360	Singapore	Male
	ZRC_BDP0014712	MK910361	Singapore	Male
	ZRC_BDP0014733	MK910362	Singapore	Male
	ZRC_BDP0016086	MK910363	Singapore	Male
	MJ13287	KU146082.1	Palau	Worker
	USNMENT01124387	KU504894.1	Philippines	Worker
*O. rixosus*	ZRC_BDP0016035	nil	Singapore	Worker
*O. pararixosus*	ZRC_BDP0012747	nil	Singapore	Worker
	ZRC_BDP0012737	nil	Singapore	Male
*O. simillimus*	ZRC_BDP0016524	nil	Singapore	Male
	ZRC_BDP0016500	nil	Singapore	Male

## Results

We found a number of reliable morphological differences between *O. malignus* and the new species *O. litoralis* sp. nov. as fully accounted below in detailed species descriptions. Comparisons of images with directly examined physical specimens revealed noticeable morphological differences between the holotype of *O. malignus* (OMH), *O. malignus* specimens from Singapore (OMSG-w) and the Philippines (OMPH-w), against workers of O. malignus var. retrolatior (OR-w). The type specimen of *O. tuberculatus* was too badly damaged and its image was thus excluded from morphological comparisons. Firstly, OMH/ OMSG-w/OMPH-w appear to have a relatively shorter scape with respect to head width, compared to OR-w (OMH SI 121, OMSG-w/OMPH-w SI 120–123; OR-w SI 127–130). Next, the gaster of OMH/OMSG-w/OMPH-w is generally uniformly dark brown in colour, but in OR-w the base of gastral tergite I is paler brown relative to the rest of the gaster. Thirdly, the petiolar spine of OR-w appears shorter and stouter relative to those of OMH/OMSG-w/OMPH-w. In view of the lack of more striking morphological differences or sympatry of the alternate forms, we conservatively infer these specimens as one and the same species with some observed variation between allopatric populations. Considering the broad distribution of *O. malignus*, more comprehensive DNA or other forms of molecular evidence from different geographic populations will be required to verify if these actually comprise of multiple cryptic species.

The COI sequences of the new species from Singapore (GenBank accession numbers: MK910364–MK910369) were nearly identical (BLAST: 100% query cover, 99% identity) to that of specimens obtained from Sarawak and identified by Matos-Maraví et al. (2018) as *Odontomachus* sp. BOR002 (GenBank accession number: KU146009). Sequences from the new species were not as closely matched (BLAST: 99% query cover, 96% identity) to that of specimens from Palau and the Philippines identified as *O. malignus* by Matos-Maraví et al. (2018) and Larabee et al. (2016) (GenBank accession numbers: KU146082.1[loc.: Palau], KU504894.1[loc.: Philippines]). The COI barcodes of 10 unidentified males, collected in (near) sympatry as the new species from the small city-state of Singapore (GenBank accession numbers: MK910354–MK910363), were almost identical (BLAST: 100% cover, 98.4% identity) to that of a specimen from Palau identified by Matos-Maraví et al. (2018) as *O. malignus* (GenBank accession number: KU146082.1). At the point of this study and based on limited DNA evidence, we tentatively identify the males collected from Pulau Semakau in Singapore as *O. malignus*, and hereinafter refer to them collectively as ‘PSM-m’. We emphasise that this identification is considered tentative as it is based on only one population; this may be subject to changes when more material comprising males from geographically allopatric populations of *O. malignus* are made available for further investigation.

**Figure 1.**
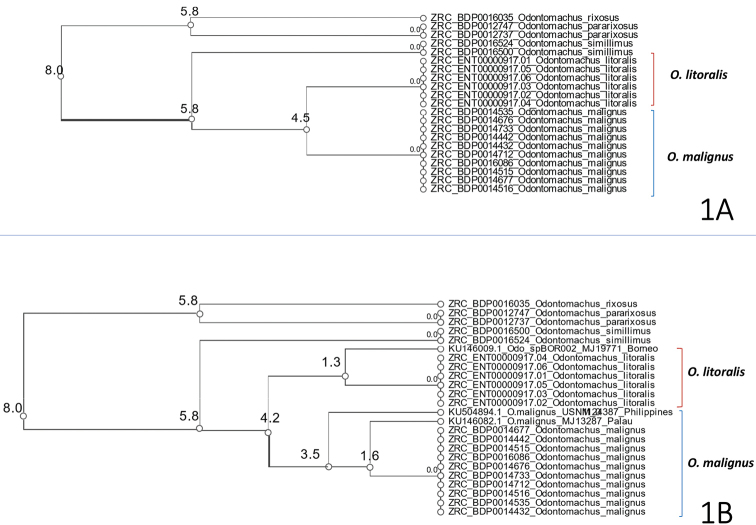
Cluster dendrogram of COI (313 bp) barcodes of *Odontomachus litoralis* and *O. malignus* from (near) sympatric populations in Singapore, specimens collected from Borneo (KU146009.1), Palau (KU146082.1), and the Philippines (KU504894.1), and additional reference barcodes of *O. rixosus*, *O. pararixosus*, and *O. simillimus*. Nodes are annotated with numbers indicating percentage (%) uncorrected *p*-distance thresholds at which sequences diverge.

In a rough sorting to putative species using objective clustering (Meier et al. 2006), COI sequences of the new species from Singapore and Borneo (ZRC_ENT00000917.01-06, KU146009.1) were divergent from sequences of specimens identified as *O. malignus* (from Singapore, Palau, and the Philippines) at an uncorrected *p*-distance threshold of 4.2% (Fig. 1). Sequences from sympatric populations (i.e., from Singapore) of *O. malignus* and the new species diverged from those of other morphologically identified congeneric species in sympatry at clustering thresholds of 8.0% (*O. rixosus*, *O. pararixosus*) and 5.8% (*O. simillimus*) respectively.

### Taxonomic accounts

#### Odontomachus malignus

Taxon classificationAnimalia

species group (preliminary)

0CFEEF28-0B28-5464-895A-6BDB07DA6FEB

##### Notes.

The following account compounds and adds on to the characters as already mentioned in Sorger and Zettel (2011). Considering the limited geographic range of specimen sampling and lack of stronger genetic evidence, this diagnosis should be deemed preliminary. The morphological and genetic bases for the treatment of this species group may be confirmed in future when more samples and comprehensive genetic information are made available for assessment. Most of the known species of the *O. infandus* group, excluding those from the Lesser Sundas, New Guinea and Fiji, were examined to clarify morphological characters that distinguish *O. malignus* and the new species from other members of the group. The *O. malignus* group is recognised by the following set of characteristics in the worker caste; those peculiar to the group are shown in italics.

##### Worker.

Head rather elongate, with CI 79–82. Temporal prominences and ocular furrow distinct. *Vertex posteriorly with a protuberance on each side of median furrow*. Mandible with a series of teeth throughout the length of its masticatory margin; apical, intercalary and subapical teeth elongate, none apically truncate, all somewhat acute; palp formula 4, 4. *Pronotal dorsum anteriorly with two or more long erect setae that are clearly discernible from short appressed or suberect hairs around them* (this condition is occasionally seen in some members of the *O. infandus* group). *Mesopleuron with anteroventral margin strongly developed*, *looking like a tubercle when seen from above.* Petiolar spine rather short, not very sharply pointed apically. First gastral tergite not flattened dorsally, without anteromedian pit. Valviceps of male genitalia lacking dorsolateral carina.

#### Odontomachus litoralis sp. nov.

Taxon classificationAnimalia

402D5710-2960-5243-985D-C833FC8F6121

http://zoobank.org/2F6BEA77-5168-493C-975B-356CC50363FC

**Figure 2.**
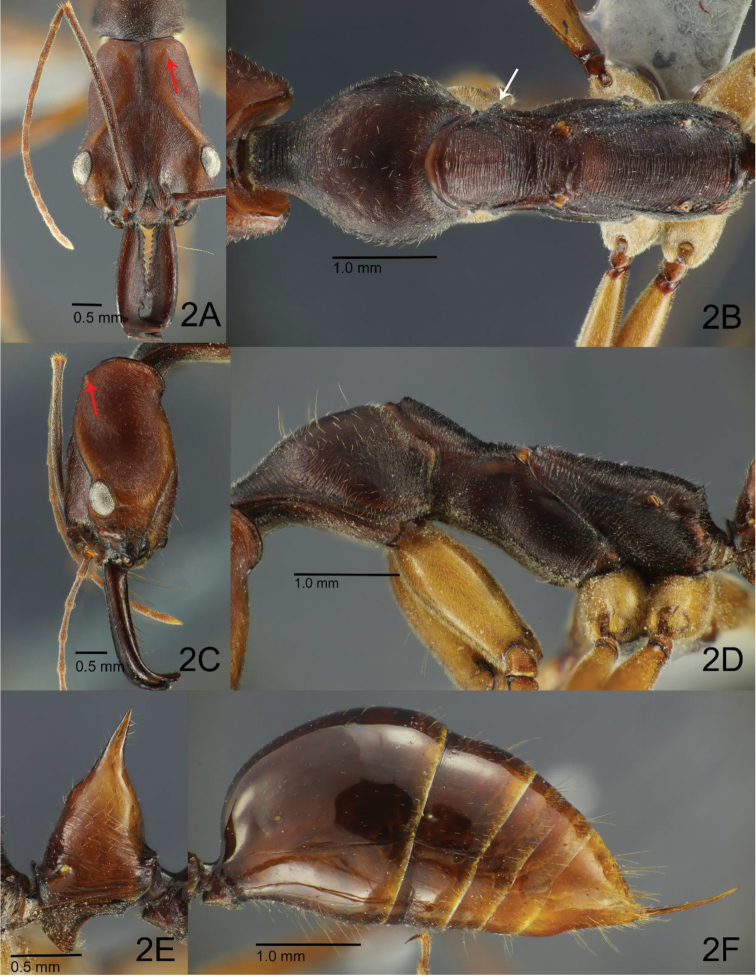
Paratype worker of *O. litoralis* sp. nov. **A** Head in full face view **B** mesosoma in dorsal view **C** head in lateral view **D** mesosoma in lateral view **E** closeup of petiole in lateral view **F** gaster in lateral view. Red arrows on **A, C** indicate protuberances on head dorsum. White arrow on **B** indicates tubercle-like projection of the strongly developed anteroventral margin of mesopleuron.

**Figure 3.**
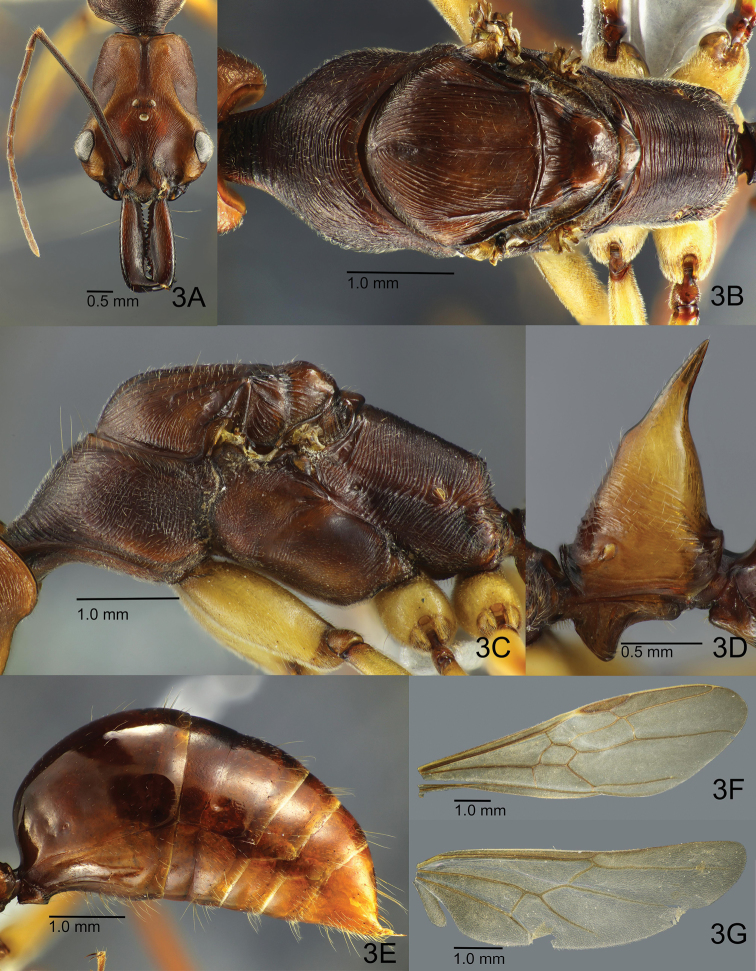
Paratype queen of *O. litoralis*. **A** Head in full face view **B** mesosoma in dorsal view **C** mesosoma in lateral view **D** closeup of petiole in lateral view **E** gaster in lateral view **F** forewing **G** hindwing.

**Figure 4.**
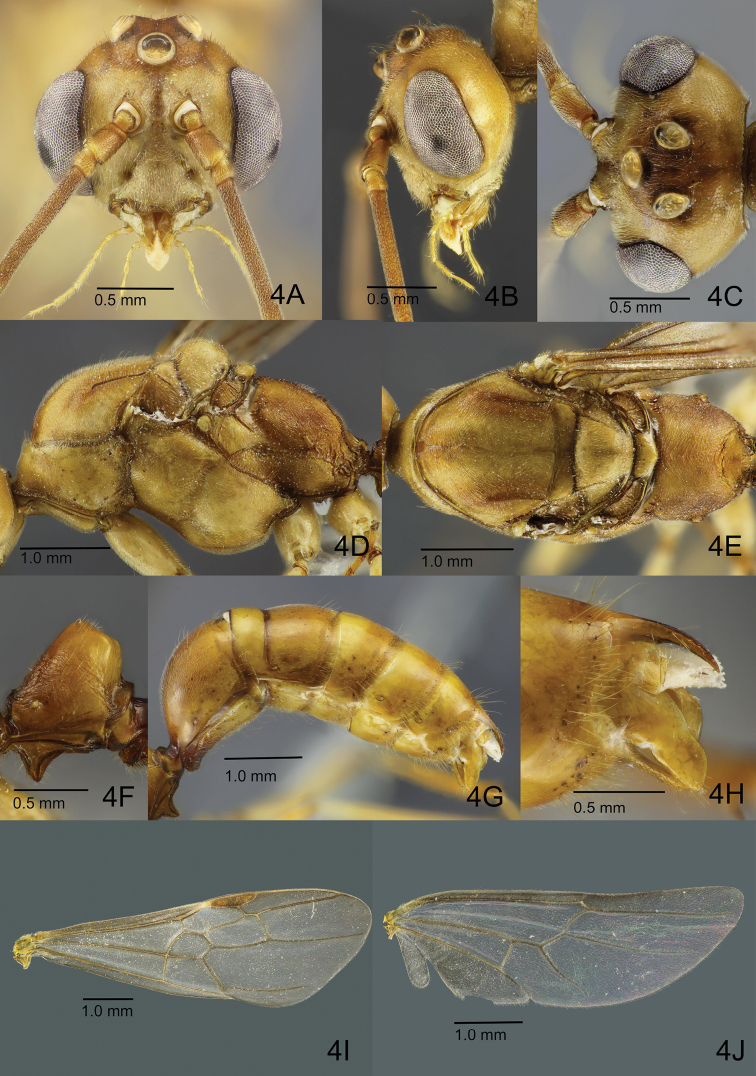
Non-type male of *O. litoralis* (ZRC_ENT00007636.1, Singapore: Sungei Buloh Wetland Reserve). **A** Head in full face view **B** head in lateral view **C** head in dorsal view **D** mesosoma in lateral view **E** mesosoma in dorsal view **F** closeup of petiole in lateral view **G** gaster in lateral view **H** closeup of gastral apex in lateral view **I** forewing **J** hindwing.

**Figure 5.**
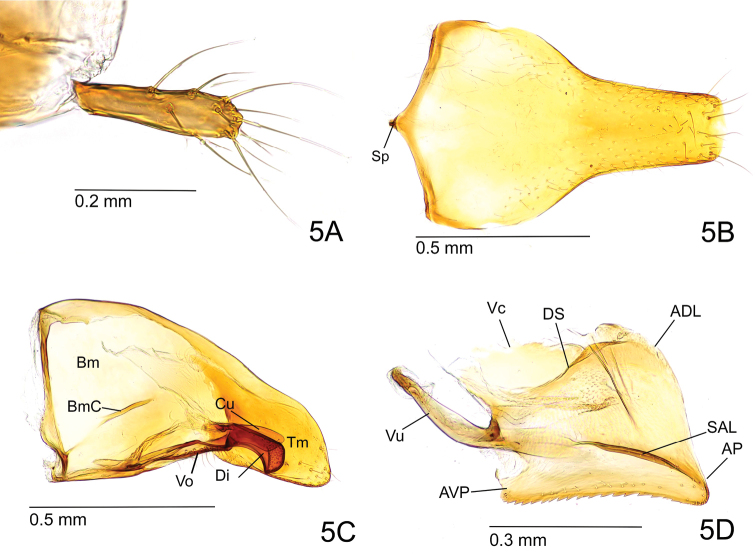
Genitalia of *O. litoralis* male, non-type (ZRC_ENT00007636.1, Singapore: Sungei Buloh Wetland Reserve). **A** Pygostyle in dorsal view **B** abdominal sternite IX in ventral view **C** paramere and volsella, right side, inner view **D** penisvalva, left side, outer view. Abbreviations: (for **B, C**) Cu – cuspis; Di – digitus; Tm – telomere; Bm – basimere; BmC – carina of lower basimere; Sp – spiculum; Vo – volsella. (for **D**) ADL – apicodorsal lobe; AP – apicoventral process; AVP – anteroventral process; DS – diagonal sclerotisation; SAL – subapical lamina; Vc – valviceps; Vu – valvura.

Figures 2 , 3 , 4 , 5

##### Types.

***Holotype*.** Worker, SINGAPORE, Sungei Buloh Wetland Reserve (1.44676N, 103.73018E), back mangrove, nest in *Thalassina* mound, 28 Mar 2018, W. Wang & M.S. Foo leg. ZRC_ENT00013883 (ZRC).

***Paratypes.*** Twenty workers, 3 queens, 1 male (NHMW, SKYC, ZRC), same data as holotype, colony no. WW-SG18-Odonto1, ZRC_ENT00000917.01–24.

##### Non-type material examined.

Singapore: 3 workers, Sungei Buloh Wetland Reserve, near Coastal Path, mangrove, nest in soil mound among roots of uprooted mangrove tree, 5 Dec 2018, W. Wang leg., colony no. WW-SG18-Odonto2, ZRC_ENT00007634 (ZRC); 4 workers, 1 queen (alate), 1 male, Sungei Buloh Wetland Reserve (1.44629N, 103.73066E), mangrove back forest, nest in abandoned *Thalassina* mound, 5 Dec 2018, W. Wang & M.S. Foo leg., colony no. WW-SG18-Odonto3, ZRC_ENT00007635 (ZRC); 4 workers, 2 males, Sungei Buloh Wetland Reserve (1.44632N, 103.73057E), mangrove back forest, nest in abandoned *Thalassina* mound, 5 Dec 2018, W. Wang, G.W. Yong & M.S. Foo leg., colony no. WW-SG18-Odonto4, ZRC_ENT00007636 (ZRC); 2 workers, Sungai [sic] Buloh Wetland Reserve, Pulau Buloh, mangrove, 7 May 2004, T.M. Leong leg. ZRC_ENT_00000779 (ZRC); 2 workers, Mandai mangroves, 1 Apr 2009, S.P. Goh and D. Pitta de Araujo leg., ZRC_ENT_00000774 (ZRC); 2 workers, Lim Chu Kang mangrove, in *Thalassina* mound, 1987, Serena Teo leg., ZRC_ENT_00000778. MALAYSIA: East Malaysia (Borneo), Sarawak, Bako (1.72382053N, 110.4451713E), ITZ mangrove forest, nest in rotten/alive roots of mangrove, 31 Mar 2012, D.M. Sorger leg., colony no. BOR12-098, MJ19771.

##### Diagnosis.

**Worker.** With features mentioned for the *O. malignus* species group. Large-sized, with moderate intranidal size variation. Head in full-face view with posterior margin weakly concave, median furrow deep and rather broad; almost entire head extensively striate to rugose, only faintly shining. Pronotum entirely with dense, fine sculpture and matte; mesonotum strigate and weakly shiny while propodeal dorsum generally more finely strigate and matte; metapleuron distinguished from propodeal dorsum by shallow longitudinal furrow; propodeal junction distinctly angular with strong transverse carina separating propodeal dorsum and declivity; posterior face of propodeum strongly marginate laterally. Basal portion of anterior face of petiolar node with strongly strigate triangular area that is rather distinctly defined; lateral face of node smooth in upper area including spine and striate in lower area. Head and petiole orange-brown; antenna, mesosoma and gaster dark reddish brown; coxae light dull-yellowish brown, femora in apical portion and tibiae orange-brown, tarsi dark brown. Legs covered with dense yellowish pubescent hairs.

**Male.** Body relatively smaller than that of worker and queen; body sculpture well-marked. Mandible subrectangular or quadrate. Furrow separating metapleural and propodeal bulges broad and deep, lined with transverse carinae along its length; petiolar node in anterior view broadly rounded apically and nearly truncate, largely microsculptured and matte, with only area around apex smooth. Maximum diameter of lateral ocellus slightly shorter than minimum distance between lateral ocellus and eye. Basal disc of abdominal sternite IX subpentagonal with broadly rounded posterolateral corners; posterior lobe of sternite IX almost as long as basal disc and slightly tapering apicad, with apical corner rounded but angulate; anterodorsal margin of valviceps strongly produced; dorsolateral carina of valviceps absent; ventral margin of valviceps broadly concave with ca. 23 denticles. Body almost entirely light dull-yellowish brown; areas around ocelli and pronotum somewhat darker; tibiae and tarsi darker than coxae and femora.

***Worker measurements.*** Holotype: EL 0.65; EW 0.50; HL 3.35; HW 2.78; IFLW 0.78; MDL 2.25; PTH 1.55; PTL 0.95; SL 3.35; WL 4.80; CI 82; MDI 67; PTHI 163; SI 122. Three paratypes, 7 non-types (*N* = 10): EL 0.55–0.65; EW 0.40–0.50; HL 2.75–3.35; HW 2.18–2.75; IFLW 0.65–0.75; MDL 1.80–2.25; PTH 1.20–1.55; PTL 0.75–0.95; SL 2.80–3.40; WL 4.10–4.90; CI 79–82; MDI 65–70; PTHI 156–168; SI 122–130.

***Queen measurements.*** Paratypes (*N* = 2): EL 0.70–0.75; EW 0.58–0.60; FWL 9.30–9.90; HL 3.35; HW 2.85–2.90; IFLW 0.85–0.90; MDL 2.20–2.30; OL 0.18–0.20; PTH 1.70–1.75; PTL 1.00–1.05; SL 3.25; WL 4.95–5.00; CI 85–87; MDI 66; PTHI 162–175; SI 112–114.

***Male measurements.*** 1 paratype, 2 non-types (*N* = 3): EL 0.75–0.80; EW 0.45–0.50; FWL 6.20–6.89; HL 1.10–1.15; HW 1.45–1.50; OL 0.20–0.25; PTH 0.70–0.75; PTL 0.75–0.80; SL 0.28–0.30; WL 3.50–3.75; CI 132–135; PTHI 93–94; SI 19.

##### Description.

**Worker.** Relatively large compared to male, with moderate variation in size (HL 2.75–3.35; WL 4.10–4.90). Head in full face view with posterior margin broadly and shallowly concave; occipital carina well-developed and dark-pigmented; median furrow deep and rather broad, with a fine longitudinal carina that is stronger anteriorly; area along each side of furrow slightly swollen; vertex area in front of occipital carina with a pair of conspicuous protuberances, each located at same distance from occipital carina and median furrow (Fig. 2A, C); temporal prominence low; extraocular furrow broad and shallow; ocular ridge narrow, widened towards median furrow; frons – taken as the medial area of head dorsum posterior to frontoclypeal suture and above antennal sockets in full-face view – clearly separated from vertex especially laterally; frontal carinae very short and weak, slightly diverging posterad; distance between anterior margin of ocular ridge and anterior margin of eye around half of major axis of eye. Mandible somewhat slender; masticatory margin distinctly dentate with 10–14 teeth/denticles, dentition being often not uniform between left and right mandibles and variable among individuals; apical tooth apically acute but often worn in aged individuals, bearing sharp intercalary tooth at mid-length; subapical tooth at least 1.5 times as long as broad, apically acute in young individuals but apex often worn and truncate in aged individuals. Palp formula 4, 4. Mesosoma relatively slender (more slender in smaller workers) compared to rest of body, in lateral view constricted at mesonotum; pronotum including its anteromedian lobe (neck) rather long, in lateral view with anterior and dorsal faces continuous and weakly convex, in dorsal view with lateral margins roundly convex; mesopleuron with conspicuous carinate anteroventral ridge, which appears like a tubercle posterior to promesonotal articulation in dorsal view (Fig. 2B); mesopleuron demarcated from mesonotum and metapleuron by more or less distinct dorsal and posterior carinae respectively; metapleuron delineated from dorsum of propodeum by a shallow longitudinal furrow spanning between basalar lobe and propodeal spiracles. Propodeum in lateral view with almost straight dorsal outline, with angular and carinate junction between dorsum and declivity, and steeply sloping posterior face with carinate lateral margins (Fig. 2D). Petiolar node in lateral view (Fig. 2E) conical, distinctly tapering apically, with anterior slope excluding apical spine almost straight to very shallowly convex, posterior slope weakly convex and steeper than anterior slope, apical spine short and apically sharply pointed, sometimes slightly curved posterad; subpetiolar process subtriangular with rounded apex, slightly longer than high, with shallowly convex anterior margin and weakly concave posterior margin. In dorsal view, gaster with tergite I large, almost as long as tergites II–IV combined; anterior face of tergite I not clearly demarcated from dorsal face, short and vertical.

Head in full face view extensively striate and only faintly shiny; frons and frontal lobes with longitudinal to weakly diverging striae, with interspaces microsculptured; ocular ridge, extraocular furrow and area anterior to vertex protuberances with weaker striae; vertex lobe distinctly strigate, temple and gena very finely microsculptured and more shiny; anterolateral portion of antennal fossa with very fine striation; entire ventral face of head covered with superficial microsculpture; median disc of clypeus superficially sculptured and shiny, with feeble striation on border with frontal lobe; mandible superficially microsculptured on dorsal and ventral faces, smooth and shiny on outer face. Pronotum in dorsal view with very fine and dense striae arranged roughly concentrically, and stronger transverse striae in anterior portion including anteromedian lobe; lateral face irregularly rugulose in anterior and posterior sections, densely microcolliculate in median portion. Mesonotum densely and finely strigate, with interspaces micropunctate; mesopleuron extensively smooth to superficially sculptured and shiny, with anterior and posterior areas transversely striate; metapleuron coarsely striate with microsculptured interspaces and weakly shining. Propodeum in dorsal view mostly with dense strigae that are weaker than those on mesonotum and metapleuron; posterior declivitous face with a few coarse transverse carinae. Anterior face of petiolar node strigate in its basal triangular area; lateral face with median section striate; remainder of anterior and lateral faces superficially microsculptured and weakly shiny; posterior face smooth and shiny. Gaster largely smooth and shining, sometimes mildly pruinose but still shiny. Legs entirely covered with microsculpture and faintly shiny.

Entire dorsal, lateral and ventral faces of head covered with numerous but scattered minute standing or decumbent hairs; frons posteriorly with pair of long erect setae, which may sometimes be lost during specimen processing; mandible covered with scattered short whitish pubescent hairs, ventral face lined with multiple long yellowish setae along masticatory margin. Dorsum of mesosoma with sparse short suberect, decumbent or appressed hairs; pronotal disc with a few sparse and long erect setae. Anterior face of petiolar node covered with short appressed or decumbent hairs; posterior face without hairs. Entire gaster with sparse fine appressed pubescence and sparser long erect setae. Legs covered with fine but dense yellowish pubescent hairs; anterior and posterior faces of procoxa and ventral faces of pro- and meso-femora with sparse erect hairs; ventral face of basal segment of protarsus with dense yellowish and stiff bristle-like hairs.

Head and petiole orange-brown, mandible somewhat darker and more reddish brown; antenna, mesosoma and gaster uniformly dark reddish brown, with apical portion of gaster yellowish; legs including coxae lighter dull-yellowish brown to orange brown; tarsus darker brown than rest of leg, but colour obscured by thick yellowish pubescence.

**Queen.** Similar to worker in general appearance, except for characters of reproductive caste. Area around ocelli not swollen; distance between lateral ocelli slightly longer than that between lateral and median ocelli, and longer than major axis of median ocellus; with head in profile median ocellus protruding dorsad; with head seen in posterodorsal view, lateral ocelli directed laterad. Mandible slightly broader relative to head than in worker; masticatory margin strongly dentate with 12–14 denticles; dentition not equal between left and right mandibles. Mesosoma in dorsal view (Fig. 3B) subcylindrical and stout, broadest around wing bases, not constricted at mesothorax. Pronotum in dorsal view with lateral margins less convex than in worker, subparallel in their posterior half; in lateral view pronotum with almost straight dorsal outline; mesonotum in dorsal view large and round; mesoscutum anteriorly roundly convex, delimited from pronotum by a deep sulcus, in posterior half margined laterally with sharp carinae that are close to forewing bases; deep furrows present between the carinae and mesoscutal disc; median line weak; notauli absent; parapsidal line distinct and reaching midlength of mesoscutal disc; mesoscutum separated from mesoscutellum by rather broad (anteroposteriorly) scutoscutellar sulcus that is laterally margined with distinct carinae; mesoscutellum much narrower and smaller than mesoscutum, broader than long, delineated from metanotum by a sharp sulcus; lateral face of pronotum posteriorly demarcated from mesopleuron by indistinct faint groove; mesopleuron distinctly divided into “anepisternum” and “katepisternum” by shallow oblique median furrow, ventrally with a carina separating main katepisternal area from narrow area right above mesocoxa (Fig. 3C). Metanotal disc much smaller than mesoscutellum, broader than long, narrowed posterad; metapleuron demarcated from mesopleuron by a fine sulcus; upper metapleural area not clearly separated from lower metapleural area, much smaller than the latter; lower metapleural area with conspicuous endophragmal pit just below upper section; spiracular sclerite distinct. Propodeum in dorsal view delineated from metanotum by distinct sulcus, gradually narrowed posterad; dorsal face evenly rounded into lateral face, which is separated from metapleuron by a shallow longitudinal furrow; posterior propodeal declivity dorsally margined with strong transverse carina, in posterior view broader than high. Petiole and gaster similar to worker (Figs 3D, E).

Head in full-face view extensively striate and similarly sculptured as in the worker, but with interspaces generally more weakly microsculptured than in the latter. Pronotum dorsally with dense transverse striae with shiny interspaces; lateral face densely and more irregularly striate, with interspaces punctate and weakly shiny; mesonotum longitudinally and rather regularly striate with interspaces punctate; “anepisternum” largely finely striate with posteroventral area smooth; oblique median furrow separating “anepisternum” from “katepisternum” weakly scrobiculate; “katepisternum” extensively minutely punctate and weakly shiny, posteriorly with coarse transverse rugae; scutoscutellar sulcus strongly scrobiculate; mesoscutellar disc largely smooth and shiny. Propodeum except for declivity transversely and regularly striate with interspaces punctate and weakly shiny; declivitous face with several widely spaced and strong transverse carinae. Anterior basal triangular area of petiolar node transversely striate with interspaces minutely punctate and weakly shiny; lateral face of node smooth and shiny except for median section with longitudinal rugae; entire petiolar spine and posterior face of node smooth to very superficially sculptured and shiny. Gaster largely smooth and shiny.

Head excluding mouthparts almost lacking erect setae; only ocellar region with pair of long erect setae; upper frons with pair of long erect hairs; entire dorsal, lateral and ventral faces of head with scattered minute standing hairs. Mandible covered with scattered fine appressed hairs; masticatory margin ventrally lined with multiple long yellowish setae. Dorsum of mesosoma with sparse short erect hairs; pronotum posteriorly bearing at least two long erect setae; propodeum with fine standing hairs mainly on dorsolateral and posterior margins but dorsum almost hairless. Petiolar spine, anterior and lateral faces of petiolar node with fine short decumbent or appressed hairs; anterior face slightly more pilose than lateral; posterior face of node without hairs. Entire gaster with scattered fine appressed pubescent hairs, and numerous but scattered long yellowish setae. Legs covered with fine but dense yellowish pubescence; ventral and lateral faces of procoxa with sparse suberect hairs; coxae and femora of mid- and hind-legs with fewer and shorter suberect hairs; ventral face of basal segment of protarsus with dense bristle-like and yellowish hairs.

Overall body colouration much as in worker. Head and petiole dark orange-brown, head sometimes more blackish in tone; mandibles slightly darker and more reddish brown; mesosoma and gaster dark reddish brown; coxae and femora yellowish to orange brown; rest of legs more reddish brown, increasingly darker towards tarsi.

Tegula distinct, elongate-ovate, much longer than broad. Wings slightly infuscate, entirely and finely setose. Forewing (Fig. 3F) with costal vein tubular to large and conspicuous pterostigma; costal, basal and subbasal cells closed; marginal cell 1, submarginal cells 1 and 2 closed; medial vein consistently strong and tubular from base to lateral wing margin. Cross-vein 1m-cu present, discal cell 1 and subdiscal cell 1 closed; cubital vein consistently tubular almost throughout entire length but fading out slightly closer to lateral wing margin. Hindwing (Fig. 3G) with jugal lobe present; basal and subbasal cells closed; basal cell roughly divided into two halves by a longitudinal spectral vein; basal one-third of radial vein (anterior margin of hindwing) lined with a series of short, stiff hamuli. With wing positioned on the same plane as body, hamuli projecting distally and dorsoventrally upwards.

**Male.** Body smaller than workers and queens (HL 1.10–1.15, WL 3.5–3.75), rather robust. Head in full-face view (Fig. 4A) much broader than long when including compound eye (hereafter simply termed ‘eye’), with roundly convex posterior margin; in lateral view dorsal outline convex around antennal insertion and vertex rather steeply sloping to occipital carina (Fig. 4B); in dorsal view strongly narrowed posterad (Fig. 4C); occipital carina thin and low, continuing anteroventrad but weakening slightly beyond midlength of lateral face of head. Clypeus posteriorly weakly demarcated from frons, gradually widened anterad, broadly produced anterad with anterior margin shallowly emarginated, with distance between anterior tentorial pits longer than that between antennal bases. Mandible reduced, subrectangular, either edentate or with 1–2 minute teeth on masticatory margin. Eye large (EL 0.75–0.80; EW 0.45–0.50), with head in full-face view bulging, breaking lateral (outer) margin of head (Fig. 4A), inner margin weakly emarginate; distance between eye and mandibular base as long as antennomere 2 (pedicel); with head in obliquely posterior view, outer margin broadly emarginate. Ocelli large, positioned on raised bases; major axis of median ocellus equal to or slightly shorter than minimum distance between lateral ocelli; maximum diameter of lateral ocellus slightly shorter than minimum distance between lateral ocellus and eye. Antenna 13-merous; antennomere 1 (scape) short, less than 1/3 as long as and slightly wider than flagellomere 1; pedicel slightly shorter than 1/2 of length of scape; flagellomeres each long, narrow and subcylindrical. Palp formula 6, 4; basal palpomere of maxillary palp very short and apical palpomere longest; labial palp with third palpomere shortest. Mesosoma elongate, much longer than high, and also longer than dorsolaterally broad. Pronotum with very short dorsal plane, in lateral view anterior slope rather steep; lateral face large, weakly widened posterad. Mesosoma in dorsal view (Fig. 4E) with mesoscutum almost as long as wide, with strongly convex anterior margin; median line weakly present in anterior half of mesoscutellar disc, in anteriormost portion branching into two short diverging carinae; notauli present as broad but shallow depressions in anterior half of mesoscutum but almost unrecognisable in its posterior half; parapsidal sulcus distinct, starting from posterolateral corner of disc to midlength of disc; scutoscutellar sulcus distinct, widened laterally; mesoscutellum much narrower than mesoscutum, broader than long, with shallow median furrow; in lateral view dorsal outline of mesoscutum largely flat with short anterior slope; mesoscutellum strongly convex dorsally; mesopleuron divided into “anepisternum” and “katepisternum” by broad but shallow furrow that widens ventrad; “katepisternum” with posteroventral carina near metacoxa. Metanotal disc small, anteroposteriorly short (narrow), well-demarcated from mesoscutellum and propodeum by sharp sulci; metapleuron divided into upper and lower areas by shallow furrow; upper area with ill-defined depression posteriorly; spiracular sclerite distinct; metapleural gland orifice occluded, but partially margined with thin walls. Propodeum delimited from metapleuron by shallow furrow, in dorsal view slightly longer than broad; propodeal junction round; posterior propodeal declivity laterally and dorsally margined with discontinuous carinae; spiracle located close to metapleuron, with its opening directed posteriorly; propodeal lobe small but distinct. Petiole (abdominal segment II) (Fig. 4F) sessile, in lateral view almost as long as high; anteriorly margined with visually-pigmented carinae; node in anterior view narrowed apicad with broadly rounded apex, in lateral view subtrapezoidal and posteriorly inclined, posterior slope shorter and steeper than anterior slope; anteroventral subpetiolar process triangular. Gastral segment I longest, in dorsal view gradually widened posterad. Legs somewhat long in proportion to mesosoma, femora longer than tibiae; meso- and meta-tibiae each with two apicoventral spurs; pretarsal claw with an inner dent; arolium relatively small compared to pretarsal claws.

Head extensively weakly and superficially sculptured, generally shiny; clypeus densely microsculptured and matte; mandible, antennal scape and pedicel superficially sculptured and shiny; entire flagellum densely microsculptured and matte. Mesosoma extensively weakly sculptured and shiny; mesoscutellum and narrow median section of metapleuron smoother and shiny; scutoscutellar sulcus longitudinally striate; shallow furrow separating metapleuron from propodeum with many strong transverse carinae across its length. Propodeum extensively very densely microsculptured, with anterior portion of dorsum strigate. Petiolar node extensively weakly sculptured, but smooth and shiny around apex. Gaster smooth to very superficially sculptured and shiny. Coxae nearly entirely smooth and shiny; femora weakly sculptured and shiny; tibiae slightly more strongly sculptured and less shiny; tarsi with dense microsculpture and matte.

Dorsum of head densely covered with short suberect hairs; longer hairs sparsely present around ocelli; ventral face of head covered with much shorter near-appressed hairs; mandible basally with some suberect hairs and apically a few longer hairs; scape with appressed hairs; pedicel and flagellum densely covered with short pubescence. Mesosoma and petiole almost entirely covered with short appressed to suberect or erect hairs; legs entirely covered with appressed pubescence. Gastral tergites and sternites with appressed to decumbent short hairs and very sparse longer hairs that are slightly denser near the posterior margin of each tergite.

Almost entire body light dull-yellowish brown; areas around ocelli and pronotum somewhat darker; areas where notauli occur present as pale-pigmented bands’; tibiae and tarsi darker than coxae and femora.

Tegula distinct, suboval, longer than broad. Wings (Fig. 4I, J) more weakly infuscate than in the queen, entirely and finely setose. Both forewing and hindwing venation and other characters much as in those in the queen.

Posterior spine of abdominal tergite VIII rather short and robust in lateral view (Fig. 4H). Pygostyle elongate-digitiform with long hairs in its apical two-thirds (Fig. 5A). Basal disc of abdominal sternite IX subpentagonal, a little broader than long; lateral margin weakly and broadly convex, slightly converging posterad with rounded posterolateral corner (Fig. 5B). Posterior lobe of sternite IX about as long as basal disc and slightly tapering apicad with apical margin almost truncated but slightly convex. Carina of lower basimere (BmC in Fig. 5C) distinct. Dorsal margin of telomere feebly concave; telomeral apex strongly produced (Fig. 5C). Ventral apex of digitus somewhat acute in lateral view (Fig. 5C). Apex of cuspis angular in lateral view. Distiventral apex relatively narrow compared to rest of valviceps in lateral view; dorsolateral carina absent; subapical lamina narrow and long; diagonal sclerotisation forming a short semi-ellipse; anterodorsal margin of valviceps strongly produced; ventral margin of valviceps broadly concave with ca. 23 denticles (Fig. 5D).

##### Distribution.

Singapore, Borneo (Sarawak).

##### Habitat.

This species inhabits the mangroves, and nests have been found in abandoned mud lobster (*Thalassina*) mounds located near back forest. It has also been found foraging near mudflats and in mangrove backforest.

##### Etymology.

The species name alludes to the littoral intertidal zone where this species is exclusively found.

##### Remarks.

The worker of the new species *O. litoralis* (OL-w) is similar to *O. malignus* workers from Singapore (OMSG-w) and the Philippines (OMPH-w), but has consistently stronger body sculpture and generally darker body colour than the latter. Some important morphological differences are as follows: 1) Propodeal junction in OL-w is strongly angulate, with the dorsum separated from the declivity by a strong transverse carina, whereas in OMSG-w and OMPH-w the propodeal junction is round with a much weaker carinate edge. 2) Propodeal declivity in OL-w is distinctly marginate laterally with raised ridges and clearly differentiated from the lateral faces of propodeum, while in OMSG-w and OMPH-w it is not distinctly margined laterally, instead rounding into the lateral faces. 3) Metapleuron in OL-w is delineated from propodeum by a shallow but broad longitudinal furrow spanning between the basalar lobe and propodeal spiracle, while in OMSG-w and OMPH-w the furrow is very weak or not recognised, thus the metapleuron is not clearly distinguished from propodeum. 4) Sculpture on the lateral face of pronotum in OL-w is always relatively coarser than that on the dorsal face and less shiny, while in OMSG-w and OMPH-w, sculpture on the lateral face of pronotum is either similar to or weaker than that on the dorsal face, with interspaces mostly smooth and shining. 5) Mandible in OL-w is longer (albeit slightly) relative to head length as compared to that of OMSG-w and OMPH-w (i.e., OL-w MDI 65–70, OMSG-/OMPH-w MDI 63–64). 6) Mesosoma is uniformly dark reddish brown in OL-w, but the pronotum has a lighter shade of brown compared to the rest of the mesosoma in OMSG-w and OMPH-w. 7) In OL-w the anterior and lateral faces of petiolar node are generally more matte with stronger superficial sculpture, while in OMSG-w and OMPH-w these are mostly smooth and shining, with only few rugae in the median section of the lateral face. Condition of sculpture on the lateral petiolar face, however, may vary between different geographic populations of the same species, for example OL-w from Sarawak has a smoother and shinier lateral petiolar face compared to OL-w from Singapore.

Males of the two species (i.e., OL-m, OMSG-m) are also morphologically similar to each other, but OL-m has consistently stronger body sculpture than OMSG-m. Males of the two species can be distinguished based on the following characters: 1) Mandible is subrectangular or quadrate in OL-m, while it is falcate and apically bluntly pointed in OMSG-m. 2) In OL-m, furrow separating the metapleuron from propodeum is broad and deep, lined with transverse carinae along its length, whereas in OMSG-m the furrow is shallow, though also lined with transverse carinae along its length. 3) Petiolar node in anterior view is apically broadly rounded in OL-m, but sharply or bluntly pointed in OMSG-m. 4) Petiolar node in OL-m is largely microsculptured and matte, with only area around the apex smooth, while in OMSG-m, the petiolar node is almost entirely smooth to superficially sculptured and shiny. 5) Maximum diameter of lateral ocellus in OL-m is slightly shorter than the minimum distance between lateral ocellus and eye, whereas it is equal to or slightly longer than the same distance for OMSG-m.

In addition, male genitalia of the two species may be differentiated using the following traits: 1) Basal disc of abdominal sternite IX is subpentagonal with broadly rounded posterolateral corners in OL-m, but with angular posterolateral corners in OMSG-m. 2) Posterior lobe of abdominal sternite IX is almost as long as basal disc, slightly tapering apicad with almost truncated apex, apical corners rounded but angulate in OL-m, but a little longer than disc, with almost parallel sides, and apex broadly and weakly convex in OMSG-m. 3) Anterodorsal margin of valviceps is strongly produced in OL-m, but weakly produced in OMSG-m. 4) Ventral margin of valviceps is broadly concave with around 23 denticles in OL-m, but undulate with ca. 33 denticles in OMSG-m.

#### Odontomachus malignus

Taxon classificationAnimalia

Smith, 1859

84198C4C-45C2-535B-90AC-A970EA8EFDA7

**Figure 6.**
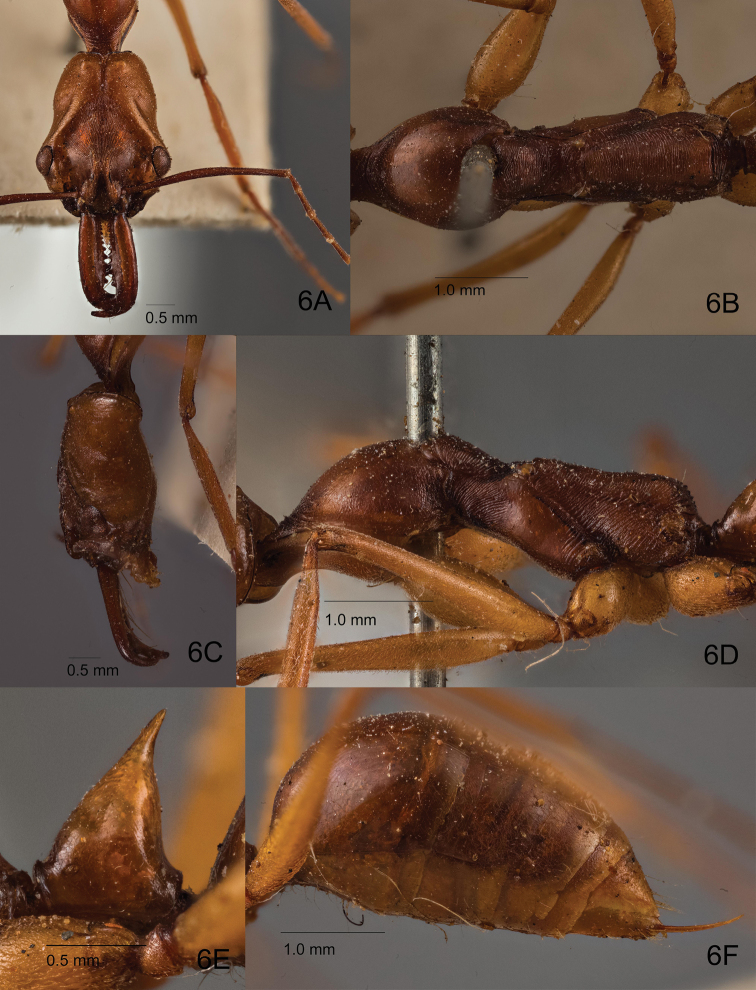
Holotype worker of *O. malignus*. **A** Head in full face view **B** mesosoma in dorsal view **C** head in lateral view **D** mesosoma in lateral view **E** closeup of petiole in lateral view **F** gaster in lateral view.

**Figure 7.**
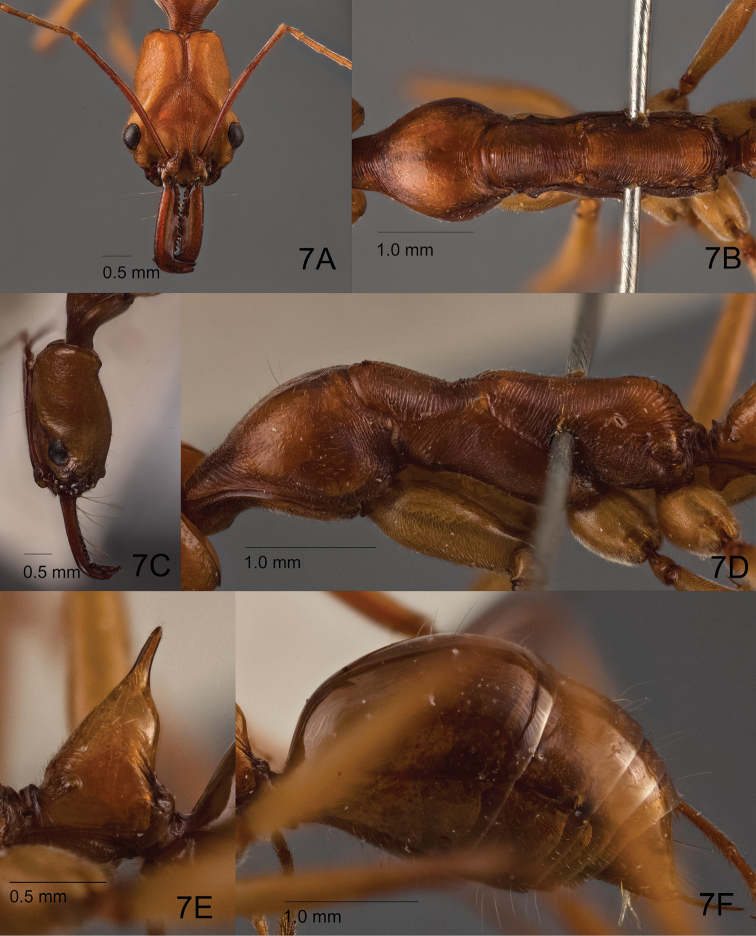
Non-type worker of *O. malignus* (ZRC_HYM0000902, Singapore: Lim Chu Kang mangrove). **A** Head in full face view **B** mesosoma in dorsal view **C** head in lateral view **D** mesosoma in lateral view **E** closeup of petiole in lateral view **F** gaster in lateral view.

**Figure 8.**
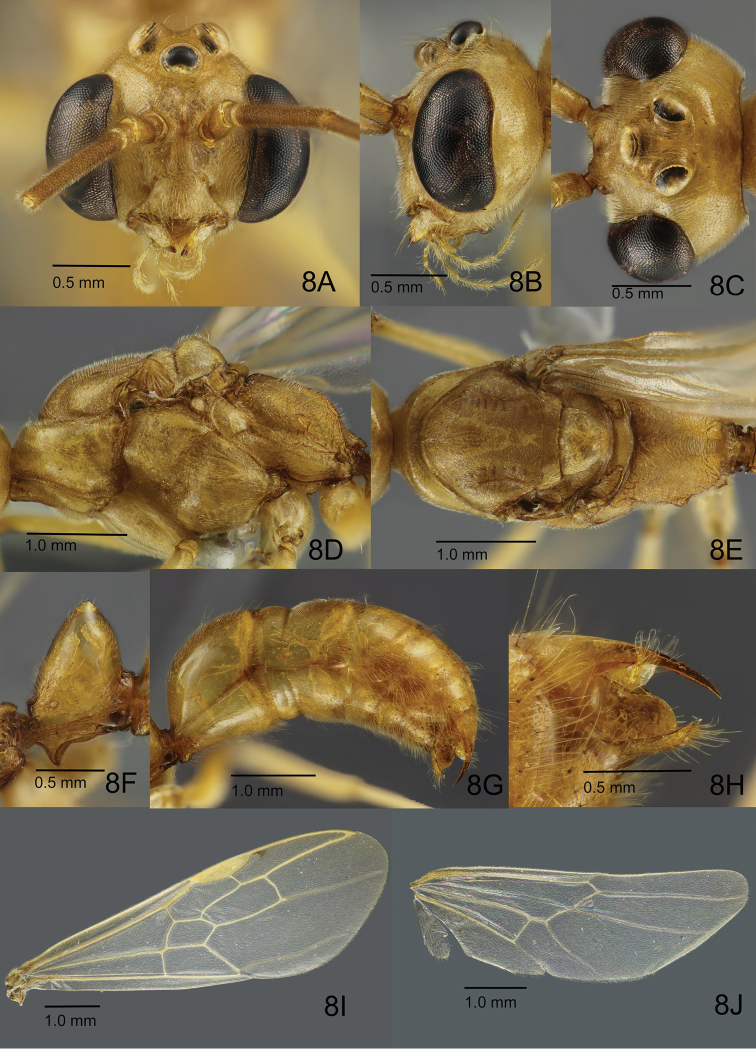
Non-type male of *O. malignus* (ZRC_BDP0014535, Singapore: Pulau Semakau, old mangrove forest). **A** Head in full face view **B** head in lateral view **C** head in dorsal view **D** mesosoma in lateral view **E** mesosoma in dorsal view **F** closeup of petiole in lateral view **G** gaster in lateral view **H** closeup of gastral apex in lateral view **I** forewing **J** hindwing.

**Figure 9.**
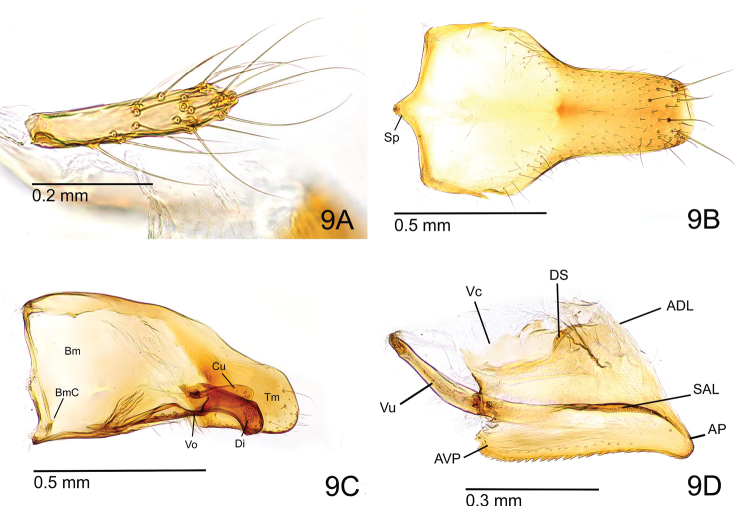
Genitalia of *O. malignus* male, non-type (ZRC_BDP0014515, Singapore: Pulau Semakau, old mangrove forest). **A** Pygostyle in dorsal view **B** abdominal sternite IX in ventral view **C** paramere and volsella, right side, inner view **D** penisvalva, left side, outer view. Abbreviations: (for **B, C**) Cu – cuspis; Di – digitus; Tm – telomere; Bm – basimere; BmC – carina of lower basimere; Sp – spiculum; Vo – volsella. (for **D**) ADL – apicodorsal lobe; AP – apicoventral process; AVP – anteroventral process; DS – diagonal sclerotisation; SAL – subapical lamina; Vc – valviceps; Vu – valvura.

Figures 6 , 7 , 8 , 9

Odontomachus malignus Smith, 1859: 144. Wilson 1959: 495; Brown 1976: 159 – 160; Olsen 2009: 11; Sorger and Zettel 2011: 155–157. Type. Holotype worker: INDONESIA, Aru Island (Smith) (A.R. Wallace) (OUMNH, ANTWEB CASENT0901334, examined)
Odontomachus tuberculatus Roger, 1861: 28 (syn. Wilson 1959) (MNHN, ANTWEB CASENT0915471, image examined).
Odontomachus malignus var. retrolatior Viehmeyer, 1914: 113 (syn. Brown 1976) (SMND, ANTWEB FOCOL0402; ZMHB, ANTWEB FOCOL1081-1082, images examined)

##### Non-type material examined.

SINGAPORE: 1 worker, Lim Chu Kang mangrove, 23 Sep (23/9-1), D.H. Murphy leg., ZRC_HYM0000902 (ZRC); 8 males, Pulau Semakau Old Fragment, 1.20664N, 103.76044E, mangrove, malaise trap SMO3, Jul-Nov 2012, M.S. Foo, P. Grooteart & J. Puniamoorthy leg., ZRC_BDP0014432/14442/14515/14516/14535/14676/14677/14712 (ZRC); 1 male, Pulau Semakau Old Fragment, 1.20489N, 103.76047E, mangrove, malaise trap SMO1, 30 Aug 2012, M.S. Foo, P. Grooteart & J. Puniamoorthy leg., ZRC_BDP0014733(ZRC); 1 male, Pulau Semakau New Fragment, 1.20125N, 103.76281E, replanted mangrove, malaise trap SMN2, 13 Dec 2012, M.S. Foo, P. Grooteart & J. Puniamoorthy leg., ZRC_BDP0016086 (ZRC). PHILIPPINES: 1 worker, Luzon, Batangas, Mabini, Mainit, 8.1.2013, C.V. Pangantihon leg., P467.

##### Diagnosis.

**Worker.** With features mentioned for the *O. malignus* species group. Body sculpture much weaker and coloration somewhat paler than in *O. litoralis*. Head in full-face view with posterior margin weakly concave; median furrow deep and rather broad; head extensively and finely (often indistinctly) striate, frontal lobes and frons with stronger striae; frontal carinae short and only slightly divergent posterad. Sculpture on pronotum much weaker than in *O. litoralis*; separation of metapleuron from propodeum indistinct; propodeal junction not strongly angulate, showing rather round transition from dorsum to declivity, declivity laterally only weakly marginate. Petiolar node extensively smooth and shiny. Head and petiole largely dark orange-brown; mandible, antenna and gaster slightly darker brown; mesosoma disc dark reddish brown.

**Male.** Body relatively smaller than that of worker, body sculpture not consistently strong. Mandible falcate, apically bluntly pointed. Furrow separating metapleuron from propodeum shallow; petiolar node in anterior view with sharply or bluntly pointed apex, almost entirely smooth to superficially sculptured and shiny. Maximum diameter of lateral ocellus equal to or slightly longer than minimum distance between lateral ocellus and eye. Body almost entirely light yellowish brown with darker greyish blotches present on head and mesosomal dorsum. Basal disc of abdominal sternite IX subpentagonal with angular posterolateral corners; anterodorsal margin of valviceps weakly produced; dorsolateral carina of valviceps absent; ventral margin of valviceps undulate with ca. 33 denticles.

***Worker measurements.*** Holotype (CASENT0901334): EL 0.55; EW 0.45; HL 2.80; HW 2.30; IFLW 0.71; MDL 1.71; PTH 1.15; PTL 0.70; SL 2.86; WL 4.09; CI 82; MDI 59; PTHI 164; SI 119. Non-types (*N* = 6; values of PTH, PTL, SL, WL, PTHI, SI obtained from 5 out of 6 specimens – previous syntype of *O. tuberculatus* excluded from measurement due to extensive damage): EL 0.46–0.60; EW 0.29–0.40; HL 2.68–3.31; HW 2.15–2.73; IFLW 0.65–0.81; MDL 1.57–1.78; PTH 1.05–1.25; PTL 0.64–0.80; SL 2.80–3.08; WL 3.99–4.33; CI 80–82; MDI 54–64; PTHI 156–164; SI 127–130.

***Male measurements.*** 3 non-types (*N* = 3): EL 0.95–0.96; EW 0.55–0.58; FWL 7.20–7.60; HL 1.30–1.36; HW 1.68–1.75; OL 0.27–0.28; PTH 0.88–0.96; PTL 0.70–0.75; SL 0.25 ; WL 3.51–3.65; CI 128–129; PTHI 123–128; SI 14–15.

##### Redescription of worker

(based on holotype, Singapore and Philippine specimens). Relatively large compared to male (HL 2.70–2.80; WL 3.95–4.00). Head in full face view (Figs 6A, 7A) longer than broad, with posterior margin weakly and broadly concave; occipital carina well-developed and dark pigmented; median furrow deep and rather broad, not much darker than rest of head dorsum; bottom of furrow with weak median carina that is stronger in its anterior portion; area along each side of furrow slightly swollen; vertex posteriorly with pair of low protuberances, each located at same distance from median furrow and occipital carina; temporal prominence low; extraocular furrow shallow; ocular ridge distinctly elevated, narrow, slightly widened toward median furrow; frons somewhat distinctly differentiated from vertex especially laterally; frontal carinae very short, diverging slightly posterad, fading out into ordinary carinae on frons; distance between anterior margin of ocular ridge and anterior margin of eye subequal to major axis of eye. Mandible rather slender; masticatory margin distinctly dentate with 11–12 denticles; dentition may not be equal between left and right mandibles; subapical tooth ca. 1.5–2 times as long as broad, tapering with somewhat acute apex (but not sharply pointed); apex sometimes worn and truncate. Palp formula 4, 4. Mesosoma in lateral view (Figs 6D, 7D) relatively slender compared to rest of body, constricted at mesonotum; pronotum including its anteromedian lobe (neck) rather long, in lateral view with gently sloping anterior face that is continuous to dorsal face, in dorsal view with roundly convex lateral margins; mesopleuron with carinate anteroventral ridge, which in dorsal view appears as a weak protuberance, (mesopleuron) demarcated by a distinct continuous sulcus dorsally from mesonotum and posteriorly from metapleuron (sulcus not margined with distinct carina); metapleuron delineated from propodeum by indistinct shallow furrow; dorsal outline of propodeum in lateral view flat to very shallowly concave; propodeal dorsum separated from declivity by weak carina with junction rounded and not strongly angulate; propodeal declivity in posterior view only weakly margined laterally. Petiolar node (Figs 6E, 7E) conical with pointed apical spine, in lateral view anterior slope almost entirely straight or weakly convex, posterior slope weakly convex and slightly steeper than anterior slope; apical spine relatively short compared to petiolar height, in frontal view gradually tapering with broad base and blunt apex, entire spine usually upright and not strongly directed posterad; subpetiolar process subtriangular with rounded apex, almost as long as high. Gastral tergite I large, in dorsal view as long as tergites II–IV combined, in lateral view with anterior slope short and vertical.

Head in full face view densely striate in most parts; frontal lobes and frons with strong striae that are longitudinal to diverging, interspaces mostly smooth and shining; anterolateral area of antennal fossa smooth and shiny; vertex with much finer striae that are often indistinct; vertex lobe, temple and lower gena with faint striation, partly micropunctate, slightly shiny; median disc of clypeus largely sculptured superficially and shiny. Dorsum and venter of mandible finely and superficially sculptured and rather shiny, lateral face smooth and shiny. Pronotum in dorsal view densely micropunctate to microreticulate with weak lustre, in lateral view irregularly striate in its anterior and posterior portions; anteromedian lobe (neck) with coarse strigae; mesonotum in dorsal view coarsely strigate with interspaces weakly microsculptured; mesopleuron densely sculptured and matte in its anterodorsal portion (upper 1/5), finely punctate but rather shiny in remaining portions, with anteriormost and posteriormost parts striate; metapleuron with sculpture similar to that of mesonotum, but with interspaces distinctly punctate. Propodeal dorsum with strigae similar to those on mesonotum; propodeal declivity with strong transverse striae that are more widely separated from each other than on propodeal dorsum. Anterior face of petiolar node mostly smooth and shiny, basally superficially sculptured with weak strigae, lateral face largely smooth and shining with median section bearing short striae, posterior face smooth and shining. Gaster mostly smooth and shining.

Head largely covered with sparse short pubescent hairs; frons posteriorly with a pair of long erect setae; mandible with scattered appressed pubescence, masticatory margin ventrally lined with multiple long yellowish setae. Dorsum of mesosoma with sparse appressed or decumbent pubescent hairs, pronotal disc with a few sparse long erect hairs. Anterior face of petiolar node with short appressed or decumbent pubescent hairs, relatively more pilose than mesosoma; posterior face of node without hairs. Entire gaster with sparse fine appressed pubescence and scattered long erect setae. Legs including coxae largely with fine but dense pubescence; ventral faces of coxae with a few sparse long erect hairs; ventral face of basal segment of protarsus with dense yellowish and stiff bristle-like hairs.

Head and petiole dark orange-brown; mandible, antenna and gaster slightly darker brown, mesosoma generally darker reddish brown; legs including coxae lighter brown and more yellowish; tarsus darker brown than rest of leg.

**Male** (based on Singapore specimens). Body smaller than worker (HL 1.30–1.36; WL 3.51–3.65), relatively robust compared to worker. Head in full-face view including eyes much broader than long, with roundly convex posterior margin (Fig. 8A); in lateral view dorsal outline convex around antennal insertion, and vertex rather steeply sloping to occipital carina (Fig. 8B); in dorsal view head strongly narrowed posterad (Fig. 8C); occipital carina thin and low, continuing anteroventrad, becoming more indistinct slightly beyond midlength of lateral face of head. Clypeus in full-face view transverse with median disc distinctly raised and flat, broadly produced anterad, with median part of anterior margin shallowly emarginate, posteriorly weakly demarcated from frons. Mandible reduced, elongate-triangular to falcate, distinctly tapering apicad with bluntly pointed and dark-pigmented apex, without teeth on masticatory margin. Eye large (EL 0.95–0.96; EW 0.55–0.58) , with head in full-face view (Fig. 8A), bulging and breaking lateral (outer) margin of head, inner margin weakly emarginate; distance between eye and mandibular base roughly as long as antennomere 2 (pedicel); with head in obliquely posterior view, outer margin broadly emarginate; ocelli large, positioned on raised bases; major axis of median ocellus equal to or slightly shorter than minimum distance between lateral ocelli; maximum diameter of lateral ocellus equal to or slightly longer than minimum distance between lateral ocellus and eye. Antenna 13-merous; antennomere 1 (scape) short, less than 1/3 as long as and slightly wider than flagellomere 1; pedicel slightly shorter than half of scape-length; flagellomeres each long, narrow and subcylindrical. Palp formula 6,4; palpomere 1 of maxillary palp very short, palpomere 6 longest; labial palp with palpomere 3 shortest. Mesosoma elongate, much longer 'much longer than high and than broad (Fig. 8D, E). Pronotum with very short dorsal plane, in lateral view anterior slope rather steep; lateral face large, weakly widened posterad, with posteroventral area distinctly depressed and concave. With mesosoma in dorsal view (Fig. 8E), mesoscutum almost as long as broad, with strongly convex anterior margin; median line only recognised in anterior half of disc, in its anterior-most portion widened anterad and margined with short carinae; notauli present as broad but shallow depressions in anterior half of mesoscutum but almost unrecognisable in its posterior half; parapsidal sulcus distinct, extending from posterolateral corner to midlength of disc; scutoscutellar sulcus distinct and widened laterally; mesoscutellum much narrower (anteroposteriorly) than mesoscutum, broader than long. In lateral view, dorsal outline of mesoscutum largely flat with short anterior slope; dorsum of mesoscutellum strongly convex; mesopleuron divided into “anepisternum” and “katepisternum” by broad and rather deep furrow that widens ventrad, leading into a concave medioventral depression; “katepisternum” with posteroventral carina near mesocoxa; metanotal disc small, short anteroposteriorly (narrow), well-demarcated from mesoscutellum and propodeum by sharp sulci; metapleuron divided into upper and lower areas by shallow furrow; upper metapleuron posteriorly with ill-defined depression; spiracular sclerite distinct; metapleural gland orifice partially margined with thin carina. Propodeum delineated from metapleuron by weak narrow sulcus, in dorsal view (Fig. 8E) slightly longer than broad; propodeal junction in lateral view rounded, posterior declivity laterally margined with weak and discontinuous carinae; transverse carina on propodeal junction obscure; propodeal spiracle located close to metapleuron, with its opening directed posteriorly; propodeal lobe small but distinct. Petiole (abdominal segment II) (Fig. 8F) sessile, about as long as high, basally margined with a pigmented carina; petiolar node tapering apicad with bluntly pointed apex; in lateral view, posterior slope shorter and steeper than anterior slope. Gaster in dorsal view broadest at segment III (abdominal segment V); gastral segment I longest, gradually widened posterad. Legs rather long in proportion to mesosoma; femora longer than tibiae; meso- and metatibiae each with two apicoventral spurs; pretarsal claw with one inner dent; arolium small.

Head extensively weakly and superficially sculptured, generally shiny; clypeus largely densely microsculptured and matte, supraclypeal area with weaker sculpture and more shiny; mandible, antennal scape and pedicel superficially sculptured and shiny; entire flagellum densely microsculptured and matte. Mesosoma extensively weakly sculptured and shiny; “katepisternum” smoother and shiny in its anterior half; mesoscutellum largely smooth and shiny; median section of metapleuron striate, or relatively smoother than other parts of metapleuron and shiny (variable among individuals); scutoscutellar sulcus longitudinally striate and shiny; shallow furrow delineating metapleuron from propodeum with many strong transverse carinae across its length. Propodeum extensively densely microsculptured, with anterior portion of dorsum strigate, dorsolateral area lined with coarse rugae. Petiolar node largely smooth and shiny. Gaster smooth to very superficially sculptured and shiny. Coxae and femora nearly entirely smooth and shiny; tibiae weakly sculptured and less shiny; tarsi densely microsculptured and weakly shining.

Dorsum of head densely covered with short suberect and erect hairs; longer standing hairs present around ocelli; clypeus and lower frons with longer hairs; ventral face of head densely covered with much shorter standing hairs; ventral edge of mandible with some long erect hairs; scape covered with short decumbent hairs, with slightly longer hairs apically; pedicel and flagellum covered with dense and short pubescence. Mesosoma and petiole almost entirely covered with short appressed to suberect or erect hairs, with a few sparse long erect hairs on pronotum; legs mostly covered with dense appressed pubescence. Gastral tergites and sternites with appressed to decumbent short hairs, and very sparse longer hairs that are largely patchily distributed but more dense nearer posterior margin of each tergite; hair covering sternites generally more dense than that on tergites.

Body almost entirely light yellowish brown; darker greyish blotches present on head and mesosomal dorsum; areas where notauli occur present as pale-pigmented bands; coxae and femora uniformly pale yellowish brown, tibiae less pale, tarsus darker brown than rest of leg, though actual colour often obscured by dense yellowish pubescence.

Tegula distinct, suboval, longer than broad. Wings not infuscate, entirely and finely setose. Forewing (Fig. 8I) with costal vein tubular to large and conspicuous pterostigma; costal, basal and subbasal cells closed; marginal cell 1, submarginal cells 1 and 2 closed; medial vein fading off distally into more spectral state towards lateral wing margin after vein 2r-m. Cross-vein 1m-cu present, discal cell 1 and subdiscal cell 1 closed; cubital vein fading off distally into more spectral state after vein 2cu-a. Hindwing (Fig. 8J) with jugal lobe present; basal and subbasal cells closed; basal cell roughly divided into two halves by longitudinal spectral vein; basal one-third of radial vein (on anterior margin of hindwing) lined with a series of short, stiff hamuli; with wing positioned on the same plane as body, hamuli directed apicad (upwards).

Posterior spine of abdominal tergite VIII rather long and slender in lateral view (Fig. 8H). Pygostyle elongate-digitiform with long hairs in its apical three-quarters (Fig. 9A). Basal disc of abdominal sternite IX subpentagonal, a little broader than long; lateral margin almost straight and slightly diverging posterad; posterolateral corner angulate (Fig. 9B). Posterior lobe of sternite IX a little longer than basal disc, slightly tapering posterad or having almost parallel sides with apical margin weakly and broadly convex. Carina of lower basimere (BmC in Fig. 9C) weak and barely recognised. Dorsal margin of telomere weakly concave; telomeral apex weakly produced (Fig. 9C). Distiventral apex of digitus right-angled in lateral view (Fig. 9C). Apex of cuspis rounded in lateral view. Distiventral apex relatively broad compared to rest of valviceps in lateral view (Fig. 9D); dorsolateral carina absent; subapical lamina narrow and long; diagonal sclerotisation forming a short semi-ellipse; anterodorsal margin of valviceps just weakly produced; ventral margin of valviceps undulate with ca. 33 denticles (Fig. 9D).

##### Distribution.

Asia – Borneo (Sarawak), Indonesia (Maluku, Sulawesi (Celebes)), Philippines, Singapore; Oceania – Palau, Papua New Guinea, Solomon Islands.

##### Habitat.

Intertidal littoral areas; workers of this species have been observed foraging in and around or emerging from exposed nest entrances in coral rubble (Brown 1976) or sheer vertical bare limestone rockfaces (Olsen 2009) along coastlines during low tides. Males were collected from mangrove forest using malaise traps, on an offshore island in the southern part of Singapore.

##### Remarks.

The workers of *O. malignus* from Singapore (OMSG-w) and the Philippines (OMPH-w) are similar to that of *O. litoralis* (OL-w), but may be distinguished from the latter by the characters listed in ‘Remarks’ under *O. litoralis*. Minor but non-negligible morphological differences between the *O. malignus* holotype (OMH), synonymised forms (e.g., *O. retrolatior* (OR-w)), OMSG-w and OMPH-w are discussed in the ‘Results’ section.

### Preliminary key to the known species of the *Odontomachus infandus* group *sensu* Brown (1976), excluding species confined to Lesser Sunda Islands, New Guinea, and Fiji, based on the worker caste. (Undescribed species are omitted; see also Sorger and Zettel (2011) and General (2018) for Philippine species.)

**Table:** 

1	Vertex posteriorly with protuberance on each side of median furrow. Mesopleuron with anteroventral margin that is strongly developed and looks like a tubercle when seen in dorsal view *O. malignus* group	**2**
–	Vertex without such protuberance. Anteroventral margin of mesopleuron less developed, not like tubercle when seen in dorsal view	**3**
2	Propodeal junction strongly angulate, with dorsum separated from declivity by strong transverse carina. Mesosoma uniformly dark reddish brown. Body sculpture stronger; lateral face of pronotum coarser than that on dorsal face and less shiny. [Singapore, Borneo]	***O. litoralis* sp. nov.**
–	Propodeal junction rounded, with more weakly carinate edge. Pronotum paler than rest of mesosoma. Body sculpture weaker; sculpture on lateral face of pronotum similar to or weaker than that on dorsal face, and interspaces mostly smooth and shiny. [Singapore, Borneo, Lesser Sundas to Oceania]	***O. malignus* Smith, F., 1859**
3	First gastral tergite entirely micropunctate, covered with dense, short appressed hairs, without flattened area and pit. Dorsum of head and almost entire mesosoma with dense erect or suberect hairs that are very short and of almost same length. Pronotal dorsum without long erect hairs. [Vietnam]	***O. silvestrii* Wheeler, 1927**
–	First gastral tergite essentially smooth and shiny (punctation, if any, very faint and sparse), anteriorly extensively flattened and often with pit corresponding to petiolar spine. Condition of short hairs on head and mesosoma variable, but hairs may be longer, appressed, curved, or much sparser depending on species. Pronotal dorsum occasionally with one or two pairs of long hairs. *O. infandus* group (s. str.)	**4**
4	Head orange-yellow to yellowish brown contrasting with dark brown to blackish mesosoma.	**5**
–	Body generally concolourous, but sometimes head, especially lateral faces, lighter than rest of body	**6**
5	Gaster dark brown. Mesosoma with dense decumbent to appressed hairs that are relatively long (generally much longer than space between hairs). Anterior face of petiole with similar hairs that are often suberect. Dorsum of head posteriorly with superficial sculpture and somewhat shiny. Lateral face and basal part of anterior face of petiole with distinct striae	***O. banksi* Forel, 1910**
–	Gaster light brown, similar to head in coloration. Hairs on mesosoma shorter (generally as long as or shorter than space between hairs). Hairs on anterior face of petiole very fine, appressed and less conspicuous. Dorsum of head posteriorly distinctly striate and matte. Petiole almost entirely smooth.	***O. alius* Sorger et Zettel, 2011**
6	Dorsum of head behind ocular prominence entirely sculptured and matte. Mesopleuron entirely striate	**7**
–	Dorsum of head behind ocular prominence essentially smooth or very faintly sculptured and shiny. Mesopleuron extensively smooth and shiny	**8**
7	Striae on pronotal dorsum essentially longitudinal, rarely weakly concentric. Petiolar spine curved backward in lateral view	***O. infandus* Smith, F., 1858**
–	Striae on pronotal dorsum more distinctly transverse. Petiolar spine almost straight in lateral view	***O. schoedli* Sorger & Zettel, 2011**
8	Body orangish light brown. Pronotal dorsum predominantly with regular transverse striae	***O. ferminae* General, 2018**
–	Body reddish brown to dark brown. Pronotal dorsum in posterior 2/3 largely with longitudinal striae	**9**
9	With petiole in dorsal view spiracle distinctly protruding laterad. Entire extraocular furrow essentially smooth with very sparse minute punctures. With mesosoma in profile, dorsal outline weakly and broadly concave	***O. scifictus* Sorger & Zettel, 2011**
–	With petiole in dorsal view spiracle not protruding laterad. Extraocular furrow predominantly smooth but with superficial striae at least partly. With mesosoma in profile, dorsal outline more straight	***O. philippinus* Emery, 1893**

## Discussion

It has long been assumed that only one species of *Odontomachus* trap-jaw ants, *O. malignus*, dominated tropical littoral habitats throughout Oceania and Southeast Asia. The discovery and validation of the new species, *O. litoralis*, existing in sympatry with *O. malignus* not only overrides this assumption, but also begs the more general question of whether we should re-assess the common notion of harsh intertidal habitats being poor in terms of diversity of terrestrial species.

For morphology, we found both species of the littoral trap-jaw ants to be similar to each other but with evident differences in structure and sculpture, especially for male bodies and genitalia between the two species. It is important to note that examined male specimens of the two species were collected from (near) sympatric populations in the small island city-state of Singapore, which has a total land area no more than 725 square kilometres (Government of Singapore 2019). Existence of the two congeneric species in near sympatry compounds the inference of morphological differences as true inter-species distinctions, and not just intra-species variation. The conclusions based on morphology and sympatry were supported by limited DNA evidence: COI barcode sequences of material from (nearly) sympatric populations in Singapore, together with allopatric populations in Borneo, Palau and the Philippines, separated into two clusters at an uncorrected *p*-distance threshold of 4.2% (Fig. 1). An objective clustering distance threshold of ca. 4% (for short-fragment COI only) has been shown to give rise to putative molecular species that are mostly congruent with morphological species, albeit with some exceptions (Wang et al. 2018a, b). The morphological similarities, in addition to small but significant COI genetic distances may also be symptomatic of very recent divergence. These are conjectures based on very limited DNA evidence; deeper sequencing of both nuclear and mitochondrial genes of specimens from populations across a broader geographic range, will be required to make inferences on genetic relatedness with greater confidence.

A notable shared feature in male genitalia of the two littoral *Odontomachus* species is the absence of a dorsolateral carina curving ventrally near the apex of the valviceps and producing the subapical lamina along the lateral apodeme; this carina is present in and deemed to be apparently unique to Nearctic and Oriental *Odontomachus* males (MacGown et al. 2014). Loss of the dorsolateral carina in male valviceps may therefore be a derived character state exclusive to the *O. malignus* species group, but this is currently a conjecture that needs further evaluation. The functional significance of this autapomorphy remains unknown. The valvura and its associated muscles have been hypothesised to be involved in controlling the movement of the valviceps apex (Boudinot 2013, Wilson et al 2016). In contrast, the dorsolateral carina of the valviceps is not associated with any muscles, and thus probably does not serve a direct mechanical function. We further hypothesise that the dorsolateral carina may also be directly involved in fitting and/or holding female genitalia during copulation; additional comparisons of female genitalia and copulatory behaviour across *Odontomachus* species will be necessary to verify this hypothesis.

**Figure 10.**
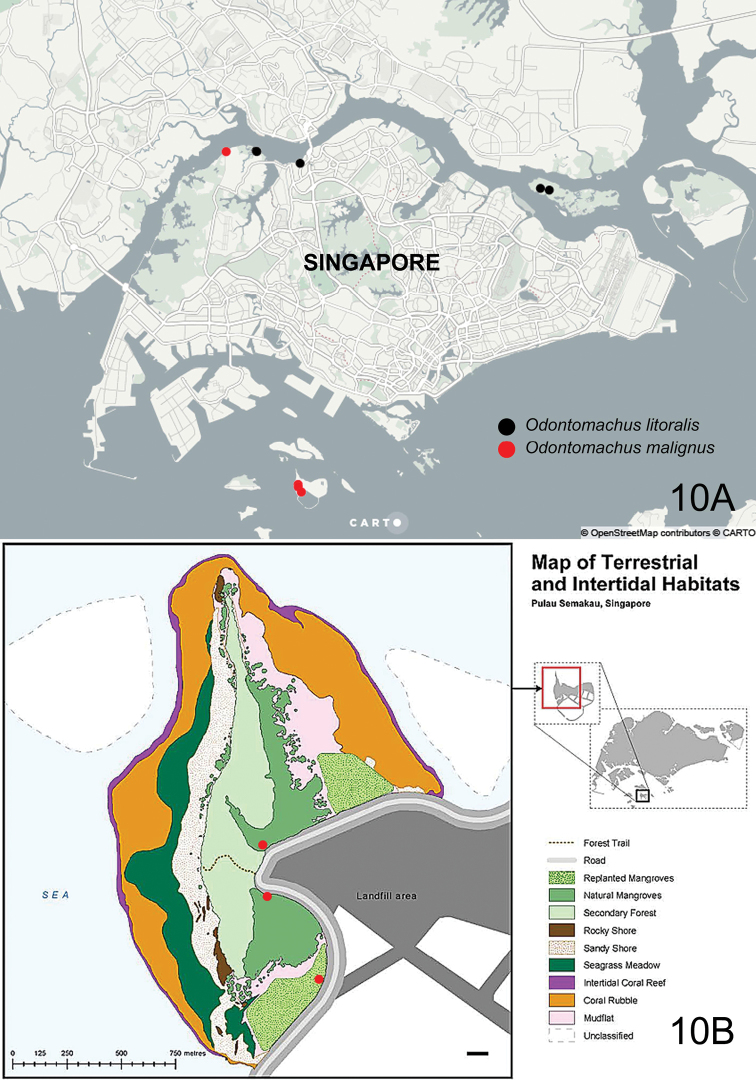
**A** Distribution of *Odontomachus malignus* and *O. litoralis* in Singapore **B** map of intertidal and terrestrial habitat types in Pulau Semakau (by Feng Yikang, for RMBR [Raffles Museum of Biodiversity Research]) and locations where *O. malignus* males were collected (indicated by red-filled circles).

The exact nesting habits of *O. malignus* remain largely unknown; past and present records indicative of its habitat type have been based on individual foraging workers and males caught in malaise traps. Limited anecdotal accounts and observations of *O. malignus* workers emerging from apparent nest entrances exposed during low tide suggest that the species possibly nests in or around coral rubble (Brown 1976), or limestone karst (Olsen 2009) next to coastlines. In contrast, *O. litoralis* has only been found nesting in abandoned mud lobster (*Thalassina* sp.) mounds further inland closer to back mangroves. This subtle stratification of the littoral habitat may suggest niche differentiation that may allow the two species to thrive sympatrically in a mosaicked habitat area. However, the *O. malignus* males used in this study were collected with malaise traps set up in mangrove forest on Pulau Semakau, an island south of Singapore mainland, not in coral rubble or limestone. The only *O. malignus* worker specimen examined from Singapore was also collected from a mangrove in the north (i.e., Lim Chu Kang) (Fig. 10A), possibly in the 1970s or 80s. No nests have been found from mangroves where the males and worker of this species were collected. These specimens may represent spill-over occurrences of *O. malignus*: actual nests of the species may be located elsewhere in nearby coral rubble or limestone rock closer to the coast. In support of this inference, the mangrove areas in Pulau Semakau where *O. malignus* males were collected are located in close proximity to extensive coral rubble surrounding the coastline (Fig. 10B).

In Singapore, *O. litoralis* nest series were sampled from northern and northeastern mangrove areas (Fig. 10A), suggesting that the two species are not exactly sympatric on a finer geographic scale, though there may be partial overlap in Lim Chu Kang mangrove. Olsen (2009) inferred that the ecological niches of *O. malignus* and its more terrestrial congener *O. simillimus* on the same limestone island could be differentiated into two distinct niches, i.e., those below and above the high tide mark respectively. In the case of *O. malignus* and *O. litoralis*, the separation of ecological niches may be more ambiguous, because *O. litoralis* nests in the mangroves are also inundated during high tide. Locating actual nesting sites of *O. malignus* in Singapore is therefore critical towards verifying our conjectures, and understanding the ecology and biology of these unique littoral trap-jaw ants.

**Figure 11.**
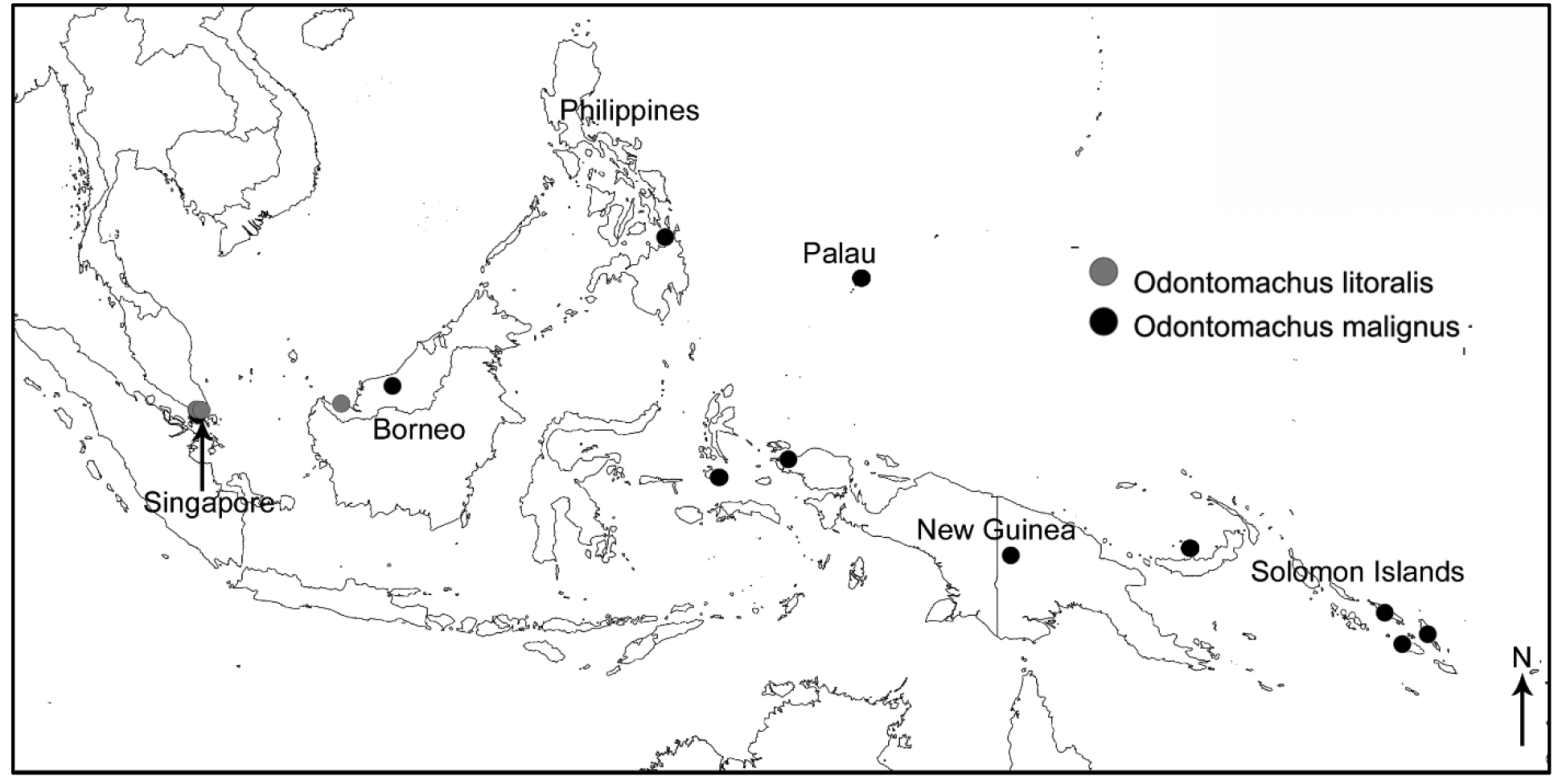
Map of current known distribution of *Odontomachus litoralis* and *O. malignus*.

Species of the *O. malignus* species group are known to be distributed throughout the Oriental and Oceanian realms sensu Holt et al. (2013); specifically, *O. malignus* has a species range that spans eastward from a westmost limit of Borneo and the Philippines in the Oriental realm, to Palau, New Guinea and finally the Solomon Islands in Oceania (Guénard et al. 2017). The discovery of *O. malignus* males in Singapore expands the existing species range further west within the Oriental realm (Fig. 11). Males collected on multiple occasions from the same locality indicate the presence of at least one established population of *O. malignus*, and not a mere transient occurrence of the species in Singapore. Human-mediated transport following increased anthropogenic activity and maritime trade between regions may explain the spread of *O. malignus* from its Melanesian origins (Matos-Maraví et al. 2018), to its current ubiquity across the Oceanian and Oriental realms, albeit restricted to intertidal zones. Another possibility is that *O. malignus* spread from Oceania to the Oriental realm by means of seasonal circulatory currents, specifically surface currents driven by yearly northeast monsoon winds, which connect the Pacific Ocean to the South China and Java Seas (Zhu et al. 2016). Fertilised queens and/or queen-right colonies of *O. malignus* may be transported by such seasonal currents while riding on ‘vessels’ such as rotten logs or branches, propagules of coastal trees, or even flotsam and other debris.

In contrast, *O. litoralis* populations have only been found in Singapore and East Malaysia (Sarawak, Borneo) (Fig. 11). The species’ more restricted distribution may probably be an artefact of inadequate sampling, given the resource limitations in this study, or it may suggest a more recent divergence from *O. malignus* in the Oriental region. The latter scenario involves the evolution of more inland-nesting habits, from strict coastlines to exploiting resources available in back mangrove. Characters necessary for survival in the harsh intertidal environment should still be maintained in *O. litoralis*, and these features may be more recently derived character states within the *O. infandus* clade. More comprehensive genetic information, other than the short COI barcodes used in this study, from populations sampled across broader geographic ranges, will be required for an accurate estimate and comparison of divergence times of both species. These are also necessary, in addition to data from multiple other congeneric species, to determine whether the littoral nesting habit is a true apomorphy, and not a case of convergent evolution. Should material from more geographically disparate populations be made available in future, inferred species boundaries in this study may even need to be re-assessed and revised. There is much more to the life histories and biology of these intriguing intertidal trap-jaw ants that awaits discovery and further study.

## Supplementary Material

XML Treatment for Odontomachus malignus

XML Treatment for Odontomachus litoralis

XML Treatment for Odontomachus malignus
